# Economic costs at age five for very preterm children with congenital anomalies: evidence from a European cohort

**DOI:** 10.1038/s41390-025-04672-z

**Published:** 2026-01-07

**Authors:** Sung Wook Kim, Veronica Siljehav, Anna-Veera Seppänen, Rolf F. Maier, Heili Varendi, Elizabeth Draper, Lazaros Andronis, Marina Cuttini, Jennifer Zeitlin, Stavros Petrou

**Affiliations:** 1 Nuffield Department of Primary Care Health Sciences, University of Oxford, Oxford, UK; 2 Department of Women’s and Children’s Health, Karolinska Institutet, Stockholm, Sweden; 3 Université Paris Cité, Inserm, INRAE, Centre for Research in Epidemiology and StatisticS (CRESS), Obstetric, Perinatal, Paediatric Life Course Epidemiology (OPPaLE) Research Team, Paris, France; 4 Children´s Hospital, Philipps University of Marburg, Marburg, Germany; 5 Tartu University Hospital, University of Tartu, Tartu, Estonia; 6 Department of Population Health Sciences, University of Leicester, Leicester, UK; 7 Warwick Clinical Trials Unit, Warwick Medical School, University of Warwick, Coventry, UK; 8 La Nostta Famiglia Association, Eugenio Medea Scientific Institute Bosisio Parini, Lecco, Italy; 9 Department of Family Medicine & Population Health (FAMPOP), Disability Studies, Faculty of Medicine & Health Sciences, University of Antwerp, Antwerp, Belgium; 10 Laboratory of Experimental Medicine and Pediatrics, Division of Neonatology, University of Antwerp, Antwerp, Belgium; 11 Study Centre for Perinatal Epidemiology Flanders, Brussels, Belgium; 12 Centre for Developmental Disabilities, Neonatal Intensive Care, Oost Limburg Hospital, Genk, Belgium; 13 Vrije Universiteit Brussel Faculteit Geneeskunde en Farmacie; Paediatric Neurology, Universitair Ziekenhuis Brussels, Brussels, Belgium; 14 Centre for Developmental Disabilities, Ghent University Hospital, Ghent, Belgium; 15 Centre for Developmental Disabilities, Leuven University Hospital, Belgium; Department of Neuropediatrics, University of Leuven, Leuven, Belgium; 16 Department of Paediatrics, Hvidovre Hospital, Copenhagen University Hospital, Hvidovre, Denmark; 17 Department of Neonatology, Hvidovre Hospital, Hvidovre, Denmark; 18 Tallinn Children’s Hospital, Tallinn; University of Tartu, Tartu, Estonia; 19 University of Tartu, Tartu University Hospital, Tartu, Estonia; 20 University of Tartu, Department of Paediatrics, Institute of Clinical Medicine, Tartu, Estonia; 21 Université Paris Cité, Inserm, INRAE, Centre for Research in Epidemiology and StatisticS (CRESS), Obstetrical Perinatal and Pediatric Epidemiology Research Team, EPOPé, Paris, France; 22 Division of Pediatrics 2, Hôpital du Bocage; INSERM CIE1, CHRU Dijon, Université de Dijon, Dijon, France; 23 Université Paris Descartes and Assistance Publique Hôpitaux de Paris, Hôpitaux Universitaire Paris Centre Site Cochin, DHU Risks in pregnancy, Service de Médecine et Réanimation néonatales de Port-Royal, Paris, France; 24 Department of Neonatology, Centre Hospitalier Intercommunal Créteil, Créteil, France; 25 Department of Neonatology, Jeanne de Flandre Hospital, Lille CHRU, Lille, France; 26 Children’s Hospital, University Hospital, Philipps University Marburg, Marburg, Germany; 27 University Medical Center, Homburg, Germany; 28 Institute of Quality Assurance Hesse, Eschborn, Germany; 29 Clinical Care and Management Innovation Research Area, Bambino Gesù Children’s Hospital, IRCCS, Rome, Italy; 30 Maternal and Child Health Institute, Marche University and Salesi Hospital, Ancona, Italy; 31 Neonatal Intensive Care Unit, Ospedale degli Infermi, Rimini, Italy; 32 Neonatal Intensive Care Unit, University Hospital S. Orsola-Malpighi, Bologna, Italy; 33 Department of Pediatrics and Neonatology, Modena University Hospital, Modena, Italy; 34 Department of Neonatology, Wilhelmina Children’s Hospital, Utrecht, the Netherlands; 35 Department of Neonatology, Poznan University of Medical Sciences, Poznan, Poland; 36 Department of Neonatology and Neonatal Infectious Diseases, Poznan University of Medical Sciences, Poznan, Poland; 37 EPIUnit-Institute of Public Health, University of Porto, Porto, Portugal; 38 Newcastle University, Newcastle upon Tyne, UK; 39 European Foundation for the Care of Newborn Infants (EFCNI), Munich, Germany; 40 Warwick Medical School, University of Warwick, Coventry, UK; 41 Université Paris Cité, Inserm, INRAE, Centre for Research in Epidemiology and StatisticS (CRESS), Obstetric, Perinatal, Pediatric Life Course Epidemiology (OPPaLE) Research Team, Paris, France

## Abstract

**Background:**

To estimate the association between very preterm birth combined with the presence of a congenital anomaly and economic costs during the fifth year of life in Europe.

**Methods:**

An economic analysis was embedded within a population-based prospective cohort study, including all infants born between 22 + 0 and 31 + 6 weeks’ gestation in 2011–2012 in 19 regions across 11 European countries. Economic costs (€, 2022 prices) during the fifth year of life were estimated for children born very preterm with (*n* = 313) and without (*n* = 3374) a congenital anomaly, and by severity of congenital anomaly. Multilevel generalised linear models explored factors associated with economic costs by anomaly severity.

**Results:**

Total mean societal costs during the fifth year of life were significantly higher among very preterm children born before 32 weeks with a congenital anomaly than those without (unadjusted mean cost difference: €2760, *p* = 0.02). A multilevel model including socioeconomic, clinical characteristics, and complications of preterm birth, showed that total mean societal costs were €3281 higher for children born before 32 weeks with congenital anomalies compared to those without (*p* < 0.001).

**Conclusion:**

Very preterm birth combined with the presence of a congenital anomaly generates significant economic costs on health and social care systems in Europe.

**Impact:**

- Very preterm birth combined with the presence of a congenital anomaly is associated with increased health and social service costs and increased societal costs during the fifth year of life.
- Additional severe neonatal morbidity is independently associated with increased costs in this population.
- Very preterm birth together with a congenital anomaly creates substantial economic burdens for health and social care systems and families five years after birth.

## Introduction

Congenital anomalies, also referred to as congenital abnormalities/malformations/disorders or birth defects, encompass structural or functional anomalies arising during intrauterine life.^1^ Congenital anomalies represent a significant global health concern, contributing to an estimated 591,000 deaths globally in 2016.^2^ The European Surveillance of Congenital Anomalies (EUROCAT) platform documented a prevalence of major congenital anomalies affecting 23.9 per 1000 births.^3^

Preterm birth, or premature birth, refers to the delivery of a baby before 37 completed weeks of gestation.^4^ Children with congenital anomalies are more likely to be born prematurely.^5^ Infants born prematurely also face several potential health problems, such as respiratory disorders, as the infant’s lungs are immature,^6^ and brain damage, such as intraventricular hemorrhage (IVH), and cystic periventricular leukomalacia (cPVL), which can have long-term consequences such as cerebral palsy,^7^ intellectual disability,^8^ and sensory (hearing/vision) impairment.^9^ Moreover, preterm infants with major congenital anomalies have nearly double the risk of neurodevelopmental impairment compared to those without congenital anomalies,^10^ and are more than three times more likely to require rehospitalisation.^10^ Mortality rates are also increased in preterm infants with congenital anomalies.

Given this, both preterm birth and congenital anomalies carry significant independent economic burdens.^11^ Complications arising from preterm birth generate significant healthcare costs, not only due to initial hospitalisations but also because of lifelong impacts.^12^ Major congenital malformations continue to be a leading reason for hospitalisation among neonates and associated costs for neonatal intensive care units.^13^

Despite the established health impacts of congenital anomalies, research on the economic consequences of congenital anomalies for preterm-born children has been less highlighted. While studies exist exploring the general economic burden of birth defects or preterm birth, they do not address the combined effects of these two conditions. For example, a study examining the economic value of lost productivity due to birth defects highlighted its significant economic impact, but did not differentiate between preterm and term births.^14^ A further study based on a cohort from California, United States, between 2008 and 2012 found that over 21,000 infants with congenital anomalies required surgery, incurring a total cost of $4.1 billion, representing 21.7% of total infant healthcare spending.^15^ This study also focused on congenital anomalies irrespective of gestational age at birth.

We aimed to contribute to the knowledge in this area by estimating the economic costs associated with congenital anomalies for very preterm (VPT)-born children at 5 years of age. To the best of our knowledge, this is the first study that explores the costs associated with VPT birth combined with the presence of a congenital anomaly using a multinational European cohort. The purpose of this study is, firstly, to estimate costs associated with congenital anomalies for VPT-born children compared to VPT-born children without a congenital anomaly and, secondly, to explore the clinical and socioeconomic factors associated with these costs.

## Methods

### Study population

Data on the characteristics of the VPT infants and their parents were obtained from the population-based Effective Perinatal Intensive Care In Europe (EPICE) cohort.^16^ EPICE included all infants born at 22 + 0 to 31 + 6 weeks gestation from 19 regions across 11 European countries: Belgium (Flanders); Denmark (Eastern Region); Estonia (entire country); France (Burgundy, Ile-de-France, and the Northern region); Germany (Hesse and Saarland); Italy (Emilia-Romagna, Lazio, and Marche); the Netherlands (Central and Eastern regions); Poland (Wielkopolska); Portugal (Lisbon and Northern region); Sweden (greater Stockholm); and the United Kingdom (East Midlands, Northern, and Yorkshire and the Humber regions). When the children were 5 years old, parents were invited to complete the Screening to improve Health In Very Preterm Infants in Europe (SHIPS) parental questionnaire. This questionnaire assessed the children’s health, development, and health-related resource utilisation in the past year, and parents’ socioeconomic status (SES), health, and wellbeing. A flow chart of the sample used in this study was presented in Appendix 1. As it can be seen from the flow chart, deaths before 5 years were excluded.

### Classification of congenital anomalies

Neonatal records were used by regional study investigators to extract details about congenital anomalies in the study population. These were coded using a predefined list of about 70 specific anomalies, with the option to document additional anomalies in free text format. Information on surgical interventions for these congenital anomalies during the neonatal period was also collected. We have defined the term “neonatal period” in our study as the period from birth up to discharge from the neonatal unit.

A team of experts, consisting of two senior neonatologists (RFM and HV), and an epidemiologist (ESD) specialised in congenital anomalies, classified the anomalies in the dataset, categorising each anomaly as mild, moderate, or severe, based on its probable impact on the child’s health status. Congenital anomalies such as oesophageal atresia and single ventricle heart physiology were classified as severe, while mild anomalies included isolated hexadactyly. Ambiguous cases, like ventricular septal defects, were considered moderate due to frequent surgical intervention, which is in line with the established hierarchy by the Report of the New England Regional Infant Cardiac Program. Unspecific malformations (e.g., “other malformation of the heart”) were excluded. Whereas the majority of answers in the questionnaires were clear, some answers remained unspecific. Therefore, congenital anomalies were subdivided into four categories: (i) category 1 = malformations which are definitely severe (for example, esophageal atresia or single left ventricle of the heart); these were classified as severe; (ii) category 2 = malformations which are definitely mild (for example isolated hexadactyly); these were classified as mild; (iii) category 3 = malformations which can be severe or mild (for example ventricular septal defect of the heart which can be severe needing surgery or mild with spontaneous closure); these were classified as moderate; and (iv) category 4 = malformations not specified (for example “other malformations of the heart”); these were excluded. Moreover, the analysis was limited to morphological malformations, excluding inborn metabolic, hematologic, or immunologic disorders.^17^ A full description of the congenital anomalies is presented in Appendix 2 and has been applied in a previous study.^17^

### Measurement of resource use and economic costs

The SHIPS parental questionnaire gathered comprehensive data on the child’s care utilisation during their fifth year of life. This included: (1) Outpatient services including visits to specialists such as paediatricians, ear, nose, and throat specialists, asthma specialists, ophthalmologists, speech therapists, psychologists, psychiatrists, and physiotherapists, and emergency department visits; (2) Hospital inpatient stays; (3) Community health and social services provided by dieticians, health visitors, occupational therapists, and early childhood intervention programmes; and (4) Additional economic impacts borne by parents due to their child’s prematurity, such as specialist equipment (e.g., wheelchairs). We also asked if the parental respondent or a family member or caregiver, such as a partner, had to take time off work because of the child’s health. The 5-year questionnaires asked parents to report how many times their child used healthcare services and other resources and asked them to write zero if the child did not use a particular service. The questionnaire has been described in a previous study^18^ and the resource use questions are reported in full in Appendix 10.

### Valuation of resource use

Due to the absence of centralised resources or publications on unit costs for various healthcare episodes or aspects in many participating countries, an iterative approach was necessary to value resource inputs. Initially, national sources or publications on unit costs, if available, were utilised. When such sources were unavailable, relevant peer-reviewed literature including published economic evaluations or cost analyses were used. Finally, cost items were validated with local health economists and project investigators.

Unit costs were converted to the reference year 2022 using a cost conversion tool^19^ as this was the latest year with available indices for the conversion tool at the time of analysis. Country-specific currencies were converted to Euros (€) using purchasing power parity (PPP) values published by the Organisation for Economic Co-operation and Development (OECD). The cost conversion tool we used employs GDP deflator values to adjust for inflation, which reflect general economic inflation rather than inflation specific to the healthcare sector. Wage compensations, representing estimated lost income due to parental care requirements, were adjusted for country variations using average wage indicators published by the OECD.^20^ We applied national average wages to value lost working days. Details on the sources and values of unit costs for each country, by resource category, can be found in Appendix 3.^16^

### Covariates

In line with previous research,^21^ the following covariates were included in multivariate models to account for their potential confounding effects: (i) Socioeconomic characteristics: mother’s country of origin, employment status, and education level; (ii) Clinical characteristics: gestational age at birth, small for gestational age (SGA) status (birthweight percentiles: <3rd, 3rd to 10th, >10th),^22^ child’s sex, mother’s age at delivery, multiplicity (twins, triplets, quadruplets) and parity (nulliparous vs. multiparous); and (iii) Complications of preterm birth: bronchopulmonary dysplasia (BPD, defined as the need for supplemental oxygen or assisted ventilation at 36 weeks’ postmenstrual age) and severe non-respiratory morbidity.^17^ In this study, severe non-respiratory morbidities included intraventricular haemorrhage grades III-IV (IVH), cystic periventricular leukomalacia (PVL), retinopathy of prematurity stages III-V (ROP) or necrotising enterocolitis needing surgery (NEC).

### Statistical analysis

Economic costs of VPT children with and without congenital anomalies were compared using means, disaggregated by gestational age category. 95% bootstrap confidence intervals were calculated. Total health and social care services costs included the costs of hospital outpatient services, hospital inpatient stays, and community health and social services. Total societal costs included all components of total health and social care services costs plus economic values for equipment needed for care, family expenses, and work losses. Additional analyses compared the costs of VPT children with mild, moderate or severe congenital anomalies with VPT children without congenital anomalies, disaggregated by gestational age at birth.

A multi-level mixed effects generalised linear model (GLM) was used to pool the results from the country-specific regressions.^23^ This multi-level model incorporates random effects at the country level. The random effects represent the variation in intercepts for countries, capturing unexplained heterogeneity beyond the fixed effects. Random effects are expressed as variances that account for cluster-specific deviations at the country level.^23^ GLM is particularly useful when the dependent variable is not normally distributed, as it uses a link function that transforms the mean of the response variable to match the range of the linear predictor.^24^ To select the most appropriate model structure for the data, the modified park test and pregibon link test were applied.^24^ Considering the results of these tests, a negative binomial distribution with a log link function was chosen for the multilevel GLM. The Poisson family was excluded as overdispersion was detected.^25^

The multi-level GLM analyses in this study were conducted with the following three model specifications for either 1) total health and social care services costs (models 1, 2 and 3) or 2) total societal costs (models 4, 5, and 6) as the dependent variable^17^: Model 1 consisted only of socioeconomic status (SES) variables as covariates. Model 2 added clinical characteristics to the SES variables. Model 3 incorporated SES variables, clinical characteristics, and the complications of preterm birth. In a similar manner, models 4, 5, and 6 were constructed with a dependent variable of total societal costs. The multi-level GLM analyses estimated costs from either a health and social care services perspective (models 1, 2 and 3) or a societal perspective (models 4, 5, and 6). Alternative model specifications were compared using Akaike Information Criterion (AIC) statistics.^26^

### Ethics

All the study regions obtained ethical approval in line with their own national legislation. The EPICE study was also approved by the French Advisory Committee on Use of Health Data in Medical Research (number 13.020). The SHIPS study was approved by the French Expert Committee for Research, Studies and Evaluations in the field of Health (number 12460bis). The French National Commission for Data Protection and Liberties approved both the EPICE (DR-2013-194) and SHIPS (DR-2018-093) studies. Parents provided informed consent for participation in the follow-up study.

## Results

Table 1 presents descriptive statistics for the study population by congenital anomaly status. The study population comprised 3687 infants, (58.3% male) with a mean gestational age at birth of 29.2 weeks and a mean birth weight of 1,235 grams. Of these infants, 313 (8.5%) had a congenital anomaly. Among the children with congenital anomalies, 52.7% were male with a mean gestational age at birth of 29.0 weeks and a mean birth weight of 1,171 grams. A flow chart of study participants by congenital anomaly status is provided in Appendix 1. Appendix 4 presents a description of the neonatal characteristics for the study population by congenital anomaly status for each country.

**Table 1:** Neonatal characteristics by congenital anomaly status.

	Congenital anomaly status		Congenital anomaly status by severity			Total
	No congenital anomaly	Congenital anomaly	Mild	Moderate	Severe	
***N***	3374	313	85	130	98	3687
**Gestational age, completed weeks, Mean (SD)**	29.2 (2.1)	29.0 (2.1)	28.1 (2.3)	28.5 (2.1)	29.1 (1.9)	29.2 (2.1)
**Birth weight (grams), Mean (SD)**	1241 (370)	1171 (365)	1101 (347)	1161 (382)	1246 (344)	1235 (370)
**Male,** ***N*** **(%)**	1803 (53.4)	165 (52.7)	38 (44.7)	71 (54.6)	56 (57.1)	1968 (58.3)
**Singleton,** ***N*** **(%)**	2292 (67.9)	217 (69.3)	57 (67.1)	89 (68.5)	71 (72.4)	2509 (68.0)
**Birthweight <3**^**rd**^ **centile for gestational age,** ***N*** **(%)**	694 (20.6)	90 (28.8)	20 (23.5)	42 (32.3)	28 (28.6)	784 (21.3)
**Birthweight 3**^**rd**^ **to 9**^**th**^ **centile for gestational age,** ***N*** **(%)**	387 (11.5)	36 (11.5)	13 (15.3)	9 (6.9)	14 (14.3)	423 (11.5)
**Birthweight ≥10**^**th**^ **centile for gestational age,** ***N*** **(%)**	2293 (68.0)	187 (59.7)	52 (61.2)	79 (60.8)	56 (57.1)	2480 (67.3)
BPD*, ***N*** **(%)**	435 (12.9)	58 (18.5)	16 (18.8)	25 (19.2)	17 (17.3)	493 (13.4)
Any severe non respiratory morbidity**, ***N*** **(%)**	330 (9.8)	48 (15.3)	19 (22.4)	19 (14.6)	10 (10.2)	378 (10.3)

^*^BPD bronchopulmonary dysplasia. This is defined as receipt of supplemental oxygen and/or ventilatory support (CPAP or mechanical ventilation) at 36 weeks of postmenstrual age.

**Any severe non-respiratory morbidity: Intraventricular haemorrhage grades III-IV (IVH), cystic periventricular leukomalacia (cPVL), retinopathy of prematurity stages III-V (ROP) or necrotising enterocolitis needing surgery (NEC).

Table 2 presents a comparison of unadjusted costs in the fifth year of life by congenital anomaly status and by gestational age at birth. Mean total societal costs differed significantly for VPT children with and without a congenital anomaly (€6180 vs €3420; mean cost difference: €2760; 95% confidence interval for the mean cost difference: €503 to €5,018). The greatest mean cost difference between children with a congenital anomaly and those without a congenital anomaly was observed in children born at 26–27 weeks (mean cost difference: €4818; 95% confidence interval for the mean cost difference: €1843 to €7793). Appendix 5 shows a comparison of costs for each cost category by congenital anomaly status. A comparison of each cost component during the fifth year of life by congenital anomaly status and by severity is presented in Appendices 5 and 6.

**Table 2:** Comparison of economic costs during the fifth year of life, by congenital anomaly status and by gestational age.

Total societal costs +	CA (*N* = 313)			No CA (*N* = 3374)		Mean difference	95% confidence interval	*P*-value
	*N*	Mean (SE)		*N*	Mean (SE)			
**Gestational age**								
**<26 weeks**	31		6704 (2298)	292	8614 (1595)	−1911	(−11664, 7842)	0.7
**26–27 weeks**	68		8381 (1997)	630	3563 (449)	4818	(1843, 7793)	0.002
**28–29 weeks**	79		6961 (1771)	866	2938 (308)	4023	(1763, 6283)	<0.001
**30–31 weeks**	135		4494 (1141)	1586	2670 (617)	1824	(−2375, 6023)	0.4
**Total**	313		6180 (827)	3374	3420 (342)	2760	(503, 5018)	0.02

CA denotes congenital anomalies.

^+^ Sum of all the cost categories.

Table 3 presents a comparison of unadjusted costs in the fifth year of life by the severity of the congenital anomaly, by gestational age at birth. The majority of mean cost estimates were higher for children with a congenital anomaly regardless of the level of its severity. The largest difference in total mean costs was observed between the severe congenital anomaly group and no congenital anomaly group (€7427 vs €3420; mean cost difference: €4007; 95% confidence interval for the mean cost difference: €35 to €7979). Appendix 6 shows a comparison of costs for each cost category by severity of congenital anomaly status. Comparison of mean costs per child during the fifth year of life by congenital anomaly status and by country is presented in Appendix 7. Multilevel GLM regressions for economic costs during the fifth year of life associated with congenital anomalies by severity are presented in Appendices 8 and 9.

**Table 3:** Comparison of economic costs during the fifth year of life, by severity of congenital anomaly status and by gestational age.

Total societal costs +	Mild CA (*N* = 85)		No CA (*N* = 3374)		Mean difference	95% confidence interval	*P*-value
	*N*	Mean (SE)	*N*	Mean (SE)			
**Gestational age**							
**<26 wks**	16	5070 (2421)	292	8614 (1595)	−3544	(−17016, 9928)	0.61
**26–27 wks**	16	17403 (6832)	630	3563 (449)	13839	(7927, 19752)	<0.001
**28–29 wks**	22	4924 (1944)	866	2938 (308)	1986	(−1859, 5831)	0.31
**30–31 wks**	31	2305 (1068)	1586	2670 (617)	−365	(−9026, 8297)	0.93
**Total**	85	6345 (1586)	3374	3420 (342)	2925	(−1331, 7182)	0.18
**Total societal costs** +	**Moderate CA (*N*** = **130)**		**No CA (*N*** = **3374)**		**Mean difference**	**95% confidence interval**	***P*-value**
	*N*	**Mean (SE)**	*N*	**Mean (SE)**			
**Gestational age**							
**<26 wks**	9	3551 (1383)	292	8614 (1595)	−5063	(−22977, 12850)	0.58
**26–27 wks**	38	4209 (917)	630	3563 (449)	645	(−2974, 4264)	0.73
**28–29 wks**	28	6808 (2674)	866	2938 (308)	3871	(378, 7363)	0.03
**30–31 wks**	55	5175 (2457)	1586	2670 (617)	2505	(−4055, 9065)	0.45
**Total**	130	5132 (1216)	3374	3420 (342)	1712	(−1738, 5162)	0.33
**Total societal costs** +	**Severe CA (*N*** = **98)**		**No CA (*N*** = **3374)**		**Mean difference**	**95% confidence interval**	***P*-value**
	*N*	**Mean (SE)**	*N*	**Mean (SE)**			
**Gestational age**							
**<26 wks**	6	15,789 (9553)	292	8614 (1595)	7175	(−14897, 29248)	0.52
**26–27 wks**	14	9396 (4576)	630	3563 (449)	5833	(−226, 11892)	0.06
**28–29 wks**	29	8653 (3844)	866	2938 (308)	5715	(2139, 9292)	0.002
**30–31 wks**	49	5115 (1363)	1586	2670 (617)	2445	(−4456, 9346)	0.49
**Total**	98	7427 (1579)	3374	3420 (342)	4007	(35, 7979)	0.05

*CA* Congenital anomalies.

^+^ Sum of all the cost categories.

Total mean health and social care services costs, as well as total societal costs, were higher for children with a congenital anomaly compared to those without a congenital anomaly in all multilevel generalised linear models (Table 4). Total health and social care services costs were higher for those with a congenital anomaly compared to those without a congenital anomaly by €1756 (*p* < 0.001) in model 3 (selected in preference to models 1 and 2 on the basis of its lower AIC statistic). Total health and social care services costs for non-native, non-European born children were lower compared to European born children by €815 (*p* < 0.001) in model 3. Factors associated with higher total health and social care services costs included male sex (€790; *p* < 0.001 in model 3), gestational age at birth <26 weeks (€1560; *p* < 0.001 in model 3), BPD (€752; *p* < 0.001 in model 3), and severe non-respiratory morbidity (€2466; *p* < 0.001 in model 3) (Table 4). Total societal costs were higher for those who had a congenital anomaly compared to those without a congenital anomaly by €3281 (*p* < 0.001) in model 6 (selected in preference to models 4 and 5 on the basis of its lower AIC statistic). Total societal costs were higher for boys than for girls (€1689; *p* < 0.001 in model 6), higher for gestational age at birth <26 weeks than 30–31 weeks (€4767; *p* < 0.001 in model 6), and higher for severe non-respiratory morbidity than no morbidity (€2333; *p* < 0.01 in model 6) (Table 4).

**Table 4:** Multi-level GLM regression of factors predicting economic costs of very preterm children during the fifth year of life by congenital anomaly status (dichotomised).

		Total health and social care services costs			Total societal costs		
		(1)^a^	(2)^b^	(3)^c^	(4)^a^	(5)^b^	(6)^c^
**Congenital anomaly (reference: no congenital anomaly)**	**Yes**	2383***	1961***	1756***	4249	3537***	3281***
		(509)	(452)	(428)	(864755)	(914)	(958)
**Employment status (reference: Employed or other situation)**	**At least one parent unemployed**	450	320	357	803	501	187
		(275)	(255)	(262)	(163342)	(531)	(582)
**Mother’s education (reference: High education ISCED 6–8)**	**Intermediate education ISCED 3–5**	212	215	175	193	203	229
		(151)	(146)	(149)	(39323)	(316)	(373)
	**Low education ISCED 0–2**	209	270	220	-8	157	244
		(210)	(210)	(211)	(1745)	(434)	(518)
**Country of birth for mothers (reference: native)**	**Non-native, European born**	−617*	−636**	−535*	−1454	−1452**	−1636**
		(247)	(238)	(262)	(295863)	(480)	(588)
	**Non-native, non-European born**	−834***	−875***	−815***	−1660	−1795***	−1834***
		(155)	(148)	(155)	(337754)	(322)	(407)
**Mother’s age at childbirth (years) (refs**.^25–34^**)**	**<25**	−97	−103	−132	−85	−125	−405
		(225)	(216)	(219)	(17314)	(453)	(521)
	**>34**	−192	−128	−127	−18	131	198
		(149)	(148)	(151)	(3573)	(328)	(392)
**Parity (reference: multiparous)**	**Nulliparous**		44	105		284	601
			(140)	(142)		(297)	(353)
**Multiples (reference: singleton)**	**Twins**		−322*	−314*		−585	−844*
			(144)	(146)		(310)	(363)
	**Triplets**		−240	−196		−269	−473
			(424)	(438)		(954)	(1099)
**Gender (reference: Female)**	**Male**		768***	790***		1603***	1689***
			(131)	(134)		(283)	(338)
**SGA (reference: >10**^**th**^ **centile)**	**<3rd centile**		332	277		-138	-430
			(218)	(217)		(528)	(641)
	**3–10**		280	174		−719	−1163*
			(170)	(170)		(371)	(476)
**Gestational age (weeks) (refs**.^30,31^**)**	**<26**		3510***	1560***		7984***	4767***
			(612)	(410)		(1457)	(1174)
	**26–27**		798***	428*		1556***	1015*
			(193)	(185)		(408)	(443)
	**28–29**		402**	279		669*	410
			(137)	(146)		(282)	(331)
**BPD (reference: no BPD)**	**Yes**			752***			1237*
				(222)			(536)
**Severe non-respiratory morbidity (reference: no severe non-respiratory morbidity)**	**Yes**			2466 ***			2333**
				(459)			(730)

^a^Model (1) and (4) : (i) Socioeconomic characteristics.

^b^Model (2) and (5) : (i) Socioeconomic characteristics + (ii) Clinical characteristics.

^c^Model (3) and (6) : (i) Socioeconomic characteristics + (ii) Clinical characteristics + (iii) Complications of preterm birth

*ISCED* International Standard Classification of Education.

Standard errors in parentheses *p < 0.05, **p < 0.01, ***p<0.001.

## Discussion

To the best of our knowledge, this is the first study that estimates the economic costs associated with congenital anomalies for children born VPT across European countries. Also, this study contributes to the knowledge by estimating costs by the severity of congenital anomalies.

This study found that congenital anomalies in VPT-born children are associated with higher costs when compared to children born VPT without a congenital anomaly, with the highest costs observed for severe congenital anomalies. The existing literature has shown higher health services use in both term born and preterm born individuals with congenital anomalies.^12,27–29^ A study in Denmark found that mothers of infants with a major congenital anomaly had greater health care utilisation across services over the 20 years following birth, although these infants were not born very preterm.^27^

This study also identified sociodemographic and socioeconomic factors that are associated with increased costs for VPT-born children with congenital anomalies in our cohort. In our study, birth to mothers who were native-born was associated with higher total health and social services costs compared to those of non-native European-born and non-native non-European-born mothers. Although there is no consensus, a systematic review covering 28 European countries and 8 other countries has found that immigrants generally use health services less than native populations, and that there are notable differences in how various immigrant sub-groups access and utilise these services.^30^

Congenital anomalies increase the risk of preterm birth,^5^ but approximately 80% of those with congenital anomalies are born at term and approximately 80% of those born preterm are moderately preterm. Therefore, our cohort of very preterm children is likely to represent a minority of children with congenital anomalies, but the incremental costs may be higher in this group than in children born at later gestational ages.

This study found that infants born at 26–27 weeks gestation with congenital anomalies incurred higher costs compared to other gestational age groups with congenital anomalies. Although no studies have examined the costs of preterm-born children with congenital anomalies by gestational age, recent research on preterm infants has shown that infants delivered at the earliest stages of pregnancy, specifically before 28 weeks gestation, incur the highest per-child medical expenses.^31,32^ Another recent study conducted with the UK cohort data found that compared to VPT and term-born groups, children born before 28 weeks gestation (extremely preterm) incurred the highest per-child cumulative hospital admission costs over an 8-year period.^33^

It should be noted that the costs associated with the presence of a congenital anomaly varied across countries, likely due to heterogeneity in health care practices. For instance, some countries do not have a ‘school nurse’ system while the UK has such a system. As a result, the number of contacts with school nurses recorded in the survey questionnaires varied significantly between countries.^16^ This is not a unique problem of this study and it has also been observed for other health care practices including dental care across Europe.^34^ Some countries, such as Sweden, Denmark and Italy have mostly government-run hospitals. On the other hand, countries such as Germany, France, and Belgium have more private hospitals and clinics.^35^ Heterogeneity not only exists in health care practices but also in the relative prices of resource inputs and personnel costs, which influenced the unit costs used in this study.

There are several strengths of this study. Firstly, this study was conducted using a large, multinational European preterm birth cohort. A large sample size was crucial to obtain a sufficient number of congenital anomaly cases for analysis. By using the EPICE/SHIPS cohort, this study enhanced statistical power, increasing the possibility of detecting statistically significant differences between children with and without congenital anomalies. Secondly, this study incorporated the economic impact of parental time off work due to care requirements for children. By including these impacts, the research provided information about wider societal costs, including the costs incurred by parents and the monetary value of work absences related to care requirements. Thirdly, this study used appropriate costing methods for a multinational cohort study. Costs in national currencies were converted to Euros (€) based on purchasing power parity (PPP) and adjusted using a cost conversion tool. This adjustment allowed us to compare costs across countries. By using a multi-level model, we attempted to control for the heterogeneity in care practices represented in the pooled data. Each country has its own health service systems that may or may not affect the independent variables and the frequency of service use. Furthermore, we internally validated our unit costs with local principal investigators in this study.

This study has some limitations that should be noted. Firstly, our classification system for congenital anomalies could benefit from further development. While two senior neonatologists and an epidemiologist with congenital anomaly expertise categorised anomalies into three severity levels, some subjectivity in classification may exist. Also, this limitation can stem from the quality of information collected from medical records at birth. In this study, the total costs were lower for moderate congenital anomalies compared to mild congenital anomalies, which is arguably contrary to expectations. Secondly, due to the heterogeneity in the availability of health care specific inflation indices across countries, general inflation indices were applied for cost adjustments. Thirdly, we did not include children that were not followed up at 5 years, and as a result, the findings of this study may be subject to sampling bias. This sampling bias, combined with the relatively small sizes of some gestational age subgroups, could have also contributed to lower cost differences for infants born at less than 26 weeks compared to those born at 26–28 weeks. Finally, parental estimates of the children’s resource use might have been subject to recall bias; however, there was no evidence that the recall bias differed between the comparison groups. In conclusion, this study explored economic costs in the fifth year of life associated with VPT birth combined with the presence of a congenital anomaly. This study sheds light on the magnitude of elevated costs even in the fifth year of life. By using a multinational European cohort, we gathered appropriate data on a previously understudied group. The information can also be used to inform clinical and budgetary service planning and parental counselling, and act as input in economic models that assess the cost-effectiveness of preventive and treatment strategies in this clinical population.

## Supplementary information

Appendix

## Data Availability

The data that support the findings of this study were used under license for the current study, and so are not publicly available.
