# Supplementary material for: Economic costs at age five for very preterm children with congenital anomalies: evidence from a European cohort
Source: Pediatr Res. 2026 Jan 7;100(3):1432–9. doi: 10.1038/s41390-025-04672-z (PMC13672051; doi:10.1038/s41390-025-04672-z)
Supplement: Supplementary file 1 [file 41390_2025_4672_MOESM1_ESM.pdf]

Appendix

Appendix 1 A flow chart of the sample used in this study

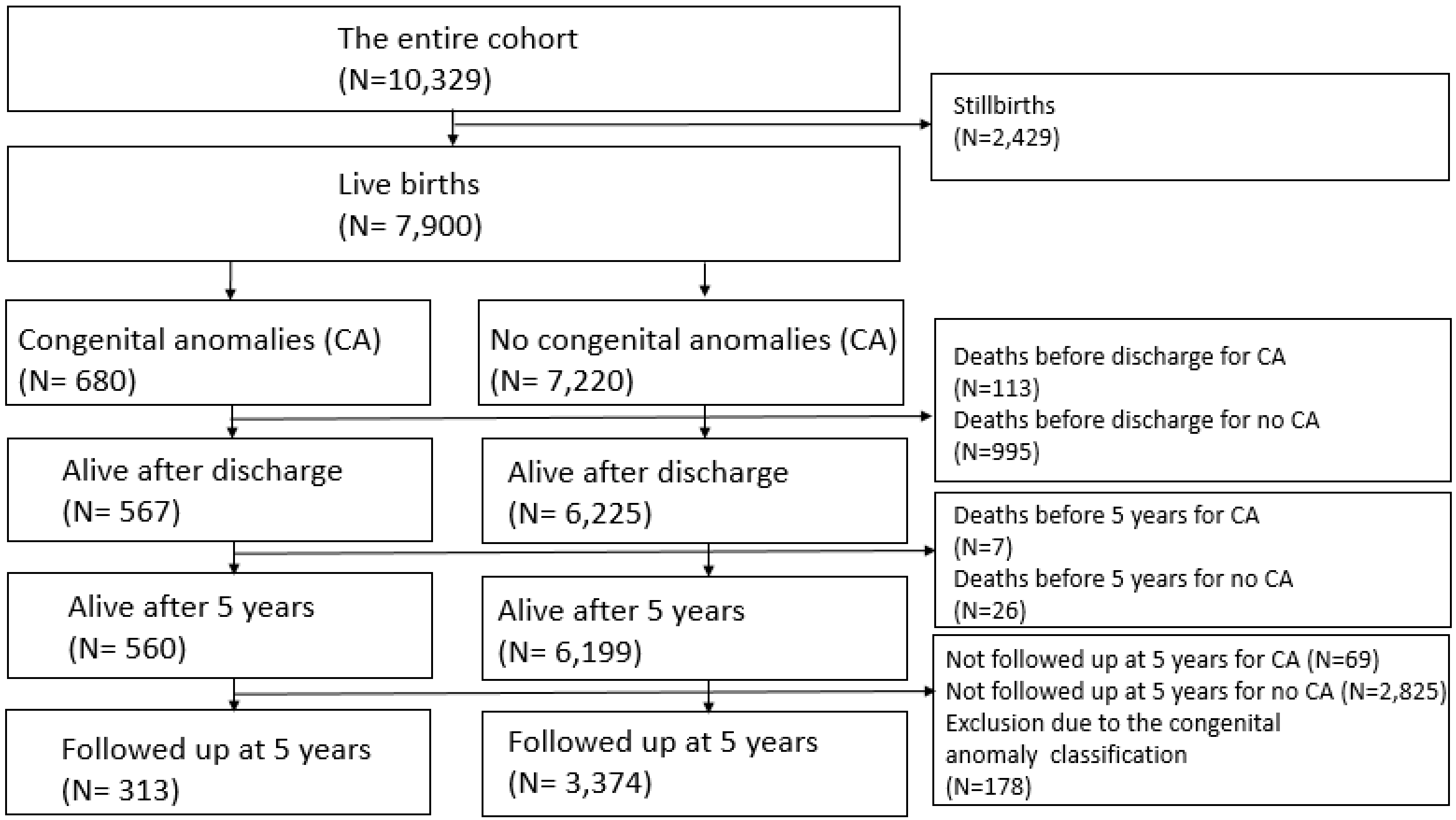

Appendix 2 Full list of congenital anomalies by severity

Organ System	Anomaly	Classification
Heart	Atrioventricular septal defect (AVSD)	Severe
Heart	Coarctation of the aorta (CoA)	Severe
Heart	Hypoplastic left heart	Severe
Heart	Pulmonary valve atresia	Severe
Heart	Aortic and mitral valves defects	Severe
Heart	Tetralogy of Fallot (ToF)	Severe
Heart	Transposition of great vessels	Severe
Heart	Tricuspidal valve atresia	Severe
Heart	Ventricular septal defect (VSD)	Moderate
Heart	Atrial septal defect (ASD)	Moderate
Heart	Pulmonary valve stenosis	Moderate
Heart	Persistent foramen ovale	Mild
Lung	Pulmonary hypoplasia	Severe
Lung	Cystic adenomatous malformation of lung	Severe
Lung	Chylotorax	Severe

Lung	Diaphragmatic hernia	Severe
Airways	Choanal atresia	Moderate
Airways	Laryngomalacia/Tracheomalacia	Moderate
Airways	Congenital malformation of larynx	Moderate
Airways	Congenital malformation of trachea and bronchus	Moderate
Mouth / Face	Macroglossia	Moderate
Mouth / Face	Cleft lip	Mild
Mouth / Face	Cleft palate	Mild
Mouth / Face	Cleft palate with cleft lip	Mild
Gastrointestinal tract	Oesophageal atresia	Severe
Gastrointestinal tract	Oesophageal atresia with trachea-oesophageal fistula	Severe
Gastrointestinal tract	Duodenal atresia/ileum atresia	Severe
Gastrointestinal tract	Duodenal stenosis/volvulus	Severe
Gastrointestinal tract	Ano-rectal atresia	Severe
Gastrointestinal tract	Hirschsprung disease	Severe

Gastrointestinal tract	Malformation of gallbladder, bile ductus and liver	Severe
Gastrointestinal tract	Gastroschisis	Severe
Gastrointestinal tract	Omphalocele	Severe
Gastrointestinal tract	Ano-rectal stenosis	Moderate
Gastrointestinal tract	Inguinal or umbilical hernia	Mild
Kidney / urinary tract	Cystic kidney disease	Severe
Kidney / urinary tract	Renal agenesis (includes Potter syndrome/facies)	Severe
Kidney / urinary tract	Renal dysplasia	Severe
Kidney / urinary tract	Ambigous genitalia / indeterminate sex	Severe
Kidney / urinary tract	Hydronephrosis	Moderate
Kidney / urinary tract	Hypospadia	Moderate
Brain / nervous system	Anencephaly	Severe
Brain / nervous system	Microcephaly	Severe
Brain / nervous system	Hydrocephalus	Severe
Brain / nervous system	Myelomeningocele	Severe
Brain / nervous system	Spina bifida	Severe

Brain / nervous system	Malformations of corpus callosum	Mild
Brain / nervous system	Cyst (arachnoid, choroid plexus)	Mild
Skull	Craniosynostosis/scaphocephalia	Severe
Limbs	Sirenomelia	Severe
Limbs	Reduction defects of lower limb	Severe
Limbs	Reduction defects of upper limb	Severe
Limbs	Hip dislocation	Severe
Limbs	Club foot / talipes equinovarus	Moderate
Limbs	Polydactyly	Mild
Limbs	Syndactyly	Mild
Skeletal system	Skeletal Dysplasia	Severe
Skeletal system	Vertebral malformation,	Severe
Skeletal system	Congenital absence of rib	Mild
Skin	Collodium Baby	Severe
Skin	Hemangioma	Mild

Skin	Aplasia cutis	Mild
Skin	Auricular, preauricular malformation (includes skin tag)	Mild
Eye	Cataract and other eye malformations	Moderate
Chromosomal anomalies	Down's syndrome (trisomy 21)	Severe
Chromosomal anomalies	Edward's syndrome (trisomy 18)	Severe
Chromosomal anomalies	Patau's syndrome (trisomy 13)	Severe
Chromosomal anomalies	Turner's syndrome	Severe
Chromosomal anomalies	Tryploidy	Severe
Complex malformations / Syndroms	Conjoined twins	Severe
Complex malformations / Syndroms	Fetal alcohol syndrome	Severe
Complex malformations / Syndroms	Warfarin syndrome	Severe
Complex malformations / Syndroms	Branchio-oculo facial syndrome	Severe
Complex malformations / Syndroms	CHARGE syndrome	Severe

Complex malformations / Syndroms	Myotonic dystrophia (Steinert)	Severe
Complex malformations / Syndroms	Ellis-van Creveld syndrome	Severe
Complex malformations / Syndroms	Simpson-Golabi-Behmel syndrome	Severe
Complex malformations / Syndroms	Di George syndrome	Severe
Complex malformations / Syndroms	Marfan syndrome	Severe
Complex malformations / Syndroms	Congenital malformation syndromes predominantly associated with short stature (Cornelia de Lange, Prader-Willi, Noonan)	Severe
Complex malformations / Syndroms	Congenital malformation syndromes predominantly affecting facial appearance (Moebius, Schinzel-Giedion)	Severe
Complex malformations / Syndroms	Tuberous sclerosis	Severe
Complex malformations / Syndroms	Sacral teratoma	Severe

Appendix 3 Sources for unit costs of resource use items

Country	Sources of unit costs
Belgium	Local PI's advice , Relevant literature [1-5]
Denmark	Kok et al. [6], Relevant literature [7-9]
Estonia	Results and costs of health care of very preterm infants in Estonia [10], Relevant literature [11]
France	Relevant literature [3, 11-17]
Germany	Relevant literature [3, 13, 18-22]
Italy	Nomenclatore specialistica 2013 [23], Relevant literature [24, 25]
Netherlands	Relevant literature [2, 3, 26-31]
Poland	[11, 32-37]
Portugal	Price consultation in NHS [38], Relevant literature [11]
Sweden	Relevant literature [3, 11, 21, 39-42]
UK	PSSRU [43], Relevant literature [44-48]

Appendix 4 Neonatal characteristics by congenital anomaly status by country

		Belgium	Denmark	Estonia	France	Germany	Italy	Netherlands	Poland	Portugal	UK	Sweden
**Total number of infants**	**No CA**	277	143	128	715	250	625	138	130	417	423	128
	**CA**	3	9	6	64	30	68	17	59	16	25	16
**Gestational age in weeks, mean (SD)**	**No CA**	29.4 (2.0)	28.9 (2.0)	29.2 (2.1)	29.4 (1.9)	29.2 (2.3)	29.4 (2.0)	28.4 (2.2)	29.4 (2.2)	29.3 (1.9)	29.0 (2.2)	28.8 (2.3)
	**CA**	28.9 (3.4)	30.0 (1.8)	29.6 (2.0)	28.8 (2.1)	29.7 (1.6)	29.1 (2.0)	27.6 (2.2)	29.1 (2.2)	29.6 (2.0)	28.8 (2.3)	28.8 (2.0)
**Male, N (%)**	**No CA**	159 (56.8)	75 (49.3)	68 (50.7)	379 (48.7)	133 (47.5)	306 (44.2)	67 (43.2)	76 (40.2)	239 (55.2)	229 (51.1)	72 (50.0)
	**CA**	0 (0.0)	3 (2.0)	5 (3.7)	34 (4.4)	12 (4.3)	36 (5.2)	8 (5.2)	30 (15.9)	11 (2.5)	16 (3.6)	10 (6.9)
**Singletons, N (%)**	**No CA**	182 (65.0)	77 (50.7)	97 (72.4)	472 (60.6)	160 (57.1)	415 (59.9)	102 (65.8)	101 (53.4)	288 (66.5)	311 (69.4)	87 (60.4)
	**CA**	3 (1.1)	5 (3.3)	6 (4.5)	42 (5.4)	22 (7.9)	45 (6.5)	13 (8.4)	47 (24.9)	10 (2.3)	14 (3.1)	10 (6.9)
**Birth weights in grams, mean (SD)**	**No CA**	1333.9 (396.6)	1209.6 (347.5)	1284.4 (365.5)	1226.7 (338.9)	1219.8 (403.1)	1245.4 (370.7)	1145.4 (365.9)	1375.9 (415.7)	1202.5 (340.3)	1231.2 (370.7)	1258.1 (407.3)
	**CA**	1130.0 (566.7)	1399.4 (398.4)	1404.5 (401.9)	1087.1 (328.4)	1336.5 (396.1)	1168.2 (338.9)	916.4 (323.3)	1209.9 (345.7)	1074.2 (315.2)	1217.5 (402.0)	1156.5 (386.4)

CA denotes congenital anomalies.

Appendix 5 Comparison of economic costs during the fifth year of life by congenital anomaly status

Cost category	CA (N=313), mean (SE)	No CA (N=3374), mean (SE)	Mean difference	95% confidence interval	P value
**Hospital outpatient services**	1556.7 (192.1)	800.5 (43.4)	756.2	(454.1,1058.2)	<0.001
**Hospital inpatient services**	1268.4 (313.1)	348.9 (41.5)	919.5	(593.4,1245.6)	<0.001
**Community health and social services**	1182.4 (405.4)	761.8 (257.3)	420.5	(-1253.6,2094.7)	0.622
**Equipment costs[1]**	160.1 (48.8)	83.0 (20.6)	77.1	(-58.9,213.1)	0.266
**Family expenses[2]**	597.5 (162.4)	302.6 (83.8)	294.9	(-253.2,843.0)	0.292
**Valuation of time work off [3]**	1414.8 (467.9)	1122.9 (106.2)	291.9	(-446.5,1030.3)	0.438
**Total societal costs+**	6179.8 (826.6)	3419.7 (342.2)	2760.1	(502.5,5017.8)	0.017

CA denotes congenital anomalies.

[1] These costs include any special equipment related to infants such as wheelchair, brace, hearing aids or shoes.

[2] These costs include any other expenses associated with child's health.

[3] These costs include salaries related to time off work

[+] Sum of all the cost categories.

Appendix 6 Comparison of economic costs during the fifth year of life by severity of congenital anomaly status

	Mild CA (N=85), mean (SE)	No CA (N=3374), mean (SE)	Mean difference	95% confidence interval	P value
**Hospital outpatient services**	2085.9 (515.4)	800.5 (43.4)	1285.4	(725.8,1844.9)	<0.001
**Hospital inpatient services**	1335.9 (794.2)	348.9 (41.5)	986.9	(418.0,1555.8)	0.001
**Community health and social services**	1707.9 (793.4)	761.8 (257.3)	946.1	(-2242.5,4134.7)	0.561
**Equipment costs[1]**	222.3 (92.7)	83.0 (20.6)	139.2	(-117.3,395.7)	0.287
**Family expenses[2]**	403.8 (144.5)	302.6 (83.8)	101.2	(-935.1,1137.5)	0.848
**Valuation of time work off [3]**	589.4 (204.5)	1122.9 (106.2)	-533.4	(-1846.9,780.0)	0.426
**Total societal costs+**	6345.1 (1585.5)	3419.7 (342.2)	2925.4	(-1330.9,7181.7)	0.178
	**Moderate CA (N=130), mean (SE)**	**No CA (N=3374), mean (SE)**	**Mean difference**	**95% confidence interval**	**P value**
**Hospital outpatient services**	1155.4 (177.4)	800.5 (43.4)	354.9	(-84.1,793.9)	0.113
**Hospital inpatient services**	732.6 (201.1)	348.9 (41.5)	383.7	(-38.3,805.6)	0.075
**Community health and social services**	359.0 (77.8)	761.8 (257.3)	-402.8	(-2973.6,2168.0)	0.759
**Equipment costs[1]**	93.3 (52.1)	83.0 (20.6)	10.3	(-196.8,217.4)	0.922
**Family expenses[2]**	360.0 (171.6)	302.6 (83.8)	57.4	(-782.3,897.2)	0.893
**Valuation of time work off [3]**	2431.4 (1104.8)	1122.9 (106.2)	1308.5	(166.2,2450.9)	0.025
**Total societal costs+**	5131.8 (1215.9)	3419.7 (342.2)	1712	(-1738.3,5162.4)	0.331
	**Severe CA (N=98), mean (SE)**	**No CA (N=3374), mean (SE)**	**Mean difference**	**95% confidence interval**	**P value**
**Hospital outpatient services**	1630.1 (345.5)	800.5 (43.4)	829.6	(317.0,1342.1)	0.002
**Hospital inpatient services**	1920.7 (673.6)	348.9 (41.5)	1571.8	(1044.1,2099.5)	<0.001
**Community health and social services**	1818.7 (1090.4)	761.8 (257.3)	1056.9	(-1926.0,4039.8)	0.487
**Equipment costs[1]**	194.8 (114.6)	83.0 (20.6)	111.8	(-128.7,352.2)	0.362
**Family expenses[2]**	1080.6 (446.9)	302.6 (83.8)	778	(-197.6,1753.6)	0.118
**Valuation of time work off [3]**	782.0 (199.2)	1122.9 (106.2)	-340.9	(-1564.5,882.7)	0.585
**Total societal costs+**	7426.8 (1578.7)	3419.7 (342.2)	4007.1	(34.7,7979.4)	0.048

CA denotes congenital anomalies.

[1] These costs include any special equipment related to infants such as wheelchairs, braces, hearing aids or shoes.

[2] These costs include any other expenses associated with the child's health.

[3] These costs include salaries related to time off work

[+] Sum of all the cost categories.

Appendix 7 Comparison of mean costs per child during the fifth year of life by congenital anomaly status and by country, by cost category

Cost category		Belgium		Denmark		Estonia		France		Germany	
		CA (N=3)	no CA (N=277)	CA (N=9)	no CA (N=143)	CA (N=6)	no CA (N=128)	CA (N=64)	no CA (N=715)	CA (N=30)	no CA (N=250)
1) Hospital outpatient services	Paediatrician	58.0 (58.0)	53.8 (8.1)	0.0 (0.0)	33.5 (7.8)	6.4 (6.4)	29.8 (6.3)	25.7 (5.5)	25.6 (1.7)	58.4 (10.1)	74.7 (6.0)
	A&E	0.0 (0.0)	5.7 (0.9)	17.6 (17.6)	24.3 (5.7)	0.0 (0.0)	15.0 (3.3)	47.2 (13.1)	70.7 (8.7)	102.8 (27.1)	103.8 (17.2)
	Neurologist	0.0 (0.0)	15.2 (3.2)	0.0 (0.0)	4.1 (1.6)	32.1 (18.4)	41.2 (30.2)	7.4 (2.8)	10.5 (6.3)	37.7 (22.1)	4.4 (1.2)
	Ear, Nose and Throat (ENT)	0.0 (0.0)	17.5 (2.2)	24.6 (17.4)	60.9 (8.5)	21.1 (12.1)	13.7 (2.9)	13.4 (3.3)	16.8 (2.7)	25.9 (8.7)	24.4 (2.8)
	Ophthalmologist	29.0 (16.7)	21.0 (6.3)	8.2 (8.2)	27.3 (5.4)	32.1 (20.9)	23.5 (3.8)	24.5 (4.3)	45.1 (5.2)	99.4 (18.1)	74.3 (6.1)
	Speech therapist	275.1 (275.1)	347.3 (78.5)	21.6 (21.6)	46.6 (16.4)	682.2 (364.2)	280.1 (68.4)	519.3 (149.3)	357.3 (41.3)	403.7 (119.7)	269.0 (39.5)
	Psychologist	0.0 (0.0)	30.6 (10.8)	16.4 (16.4)	21.1 (9.4)	1338.2 (1338.2)	88.9 (26.8)	40.5 (22.6)	42.1 (8.2)	27.9 (17.9)	33.8 (29.0)
	Psychiatrist	0.0 (0.0)	2.0 (0.8)	0.0 (0.0)	0.0 (0.0)	6.4 (6.4)	0.6 (0.6)	35.1 (16.6)	32.7 (15.6)	3.0 (2.1)	2.2 (1.8)
	Physiotherapist	0.0 (0.0)	240.8 (41.2)	0.0 (0.0)	151.4 (66.3)	390.4 (324.9)	98.4 (33.3)	404.9 (129.7)	176.7 (24.9)	285.8 (90.3)	57.0 (19.5)
	Respiratory specialist	0.0 (0.0)	7.2 (2.0)	0.0 (0.0)	5.7 (2.4)	6.4 (6.4)	10.5 (2.3)	7.7 (3.5)	9.1 (1.4)	8.4 (3.7)	1.6 (0.6)
1)Total Hospital outpatient services		362.1 (238.9)	741.1 (106.0)	88.4 (53.4)	375.0 (78.2)	2515.4 (1527.6)	601.7 (107.3)	1125.6 (249.9)	786.7 (64.2)	1052.9 (173.2)	645.2 (65.5)
2) Hospital inpatient services		0.0 (0.0)	225.3 (55.1)	0.0 (0.0)	143.5 (107.3)	138.1 (138.1)	41.6 (15.2)	330.5 (155.7)	212.8 (39.5)	2340.7 (1230.5)	623.0 (114.7)

**3) Community health and social services**	**GP**	67.7 (53.8)	75.2 (5.7)	47.8 (19.9)	88.9 (9.0)	44.8 (20.1)	28.1 (3.3)	52.1 (7.2)	75.2 (5.7)	30.8 (10.4)	24.7 (5.0)
	**Dietician**	0.0 (0.0)	1.4 (0.8)	0.0 (0.0)	4.9 (2.2)	6.4 (6.4)	0.6 (0.6)	0.0 (0.0)	0.4 (0.4)	3.3 (2.3)	1.0 (0.5)
	**Health visitor**	0.0 (0.0)	0.5 (0.3)	0.0 (0.0)	6.6 (2.4)	4.2 (4.2)	23.6 (7.2)	0.0 (0.0)	0.0 (0.0)	9.6 (6.7)	1.6 (1.1)
	**School Nurse**	17.0 (17.0)	15.1 (5.7)	0.0 (0.0)	0.0 (0.0)	8.4 (7.7)	0.7 (0.2)	0.0 (0.0)	0.0 (0.0)	0.0 (0.0)	0.3 (0.2)
	**Occupational therapist**	0.0 (0.0)	118.7 (43.1)	0.0 (0.0)	38.2 (16.5)	156.2 (100.4)	10.0 (5.4)	1.9 (1.9)	58.5 (40.6)	550.9 (161.6)	285.9 (47.7)
	**Early childhood intervention**	0.0 (0.0)	48.1 (29.5)	8.9 (8.9)	68.7 (58.2)	0.0 (0.0)	19.2 (17.9)	0.0 (0.0)	0.0 (0.0)	127.9 (56.6)	59.7 (16.2)
	**Other costs**	0.0 (0.0)	3.2 (0.7)	8.9 (8.9)	5.6 (1.7)	37.5 (11.9)	19.5 (2.7)	4.3 (2.2)	0.3 (0.2)	6.6 (2.7)	6.3 (1.2)
**3) Total Community health and social services**		84.7 (70.7)	262.3 (54.2)	65.6 (27.0)	213.0 (60.4)	257.5 (110.9)	101.7 (20.6)	58.2 (8.5)	134.5 (40.9)	729.2 (188.8)	379.4 (52.8)
**4) Equipment costs[1]**		78.9 (50.9)	51.0 (24.4)	655.6 (655.6)	370.2 (131.5)	93.6 (57.1)	43.3 (19.7)	17.0 (6.0)	28.2 (5.6)	42.7 (23.4)	21.7 (7.9)
**5) Family expenses[2]**		0.0 (0.0)	74.1 (25.2)	65.6 (65.6)	175.3 (54.5)	278.6 (278.6)	269.9 (168.3)	62.6 (43.2)	34.8 (12.2)	198.1 (93.2)	31.4 (12.3)
**6) Valuation of time work off [3]**		609.0 (609.0)	1842.3 (514.0)	0.0 (0.0)	2112.1 (902.3)	2915.0 (2527.0)	1063.3 (212.2)	284.3 (99.9)	418.0 (95.9)	1045.0 (269.5)	666.3 (281.4)
**7) Total societal costs+**		1134.6 (629.7)	3196.0 (587.9)	875.0 (661.3)	3389.1 (953.5)	6198.2 (2554.6)	2121.5 (326.0)	1878.3 (404.3)	1615.0 (147.5)	5408.7 (1457.8)	2367.1 (331.1)

(Continued)

Cost category		Italy		Netherlands		Poland		Portugal		UK		Sweden	
		CA (N=68)	no CA (N=625)	CA (N=17)	no CA (N=138)	CA (N=59)	no CA (N=130)	CA (N=16)	no CA (N=417)	CA (N=25)	no CA (N=423)	CA (N=16)	no CA (N=128)
**1) Hospital outpatient services**	**Paediatrician**	107.1 (14.8)	91.9 (8.4)	6.0 (3.2)	16.9 (4.1)	613.6 (153.2)	584.4 (53.4)	65.1 (30.2)	65.1 (5.6)	386.7 (129.6)	261.8 (46.4)	659.2 (378.6)	181.0 (33.2)
	**A&E**	41.9 (13.6)	29.3 (2.8)	43.8 (19.7)	48.5 (10.5)	18.4 (5.2)	10.2 (1.9)	89.3 (22.4)	89.4 (6.3)	185.7 (68.8)	148.1 (27.0)	154.1 (78.5)	159.6 (34.9)
	**Neurologist**	11.4 (2.8)	6.8 (1.0)	4.3 (4.3)	4.7 (3.0)	104.0 (25.2)	50.0 (14.2)	5.9 (5.9)	7.5 (1.6)	2.1 (1.5)	1.0 (0.2)	197.1 (91.7)	56.3 (18.4)
	**Ear, Nose and Throat (ENT)**	12.5 (2.4)	13.5 (2.1)	29.9 (21.6)	24.8 (5.1)	27.9 (9.0)	46.5 (10.7)	35.5 (11.0)	35.9 (3.3)	46.2 (14.4)	22.6 (3.1)	131.7 (51.2)	65.8 (17.2)
	**Ophthalmologist**	20.9 (4.3)	26.0 (3.7)	20.5 (11.4)	59.6 (23.9)	81.3 (19.2)	56.5 (10.2)	29.6 (12.9)	38.1 (4.5)	29.1 (7.9)	24.0 (2.2)	214.3 (69.3)	136.2 (36.4)
	**Speech therapist**	145.4 (36.2)	76.1 (9.6)	90.9 (72.5)	163.8 (46.8)	1436.1 (307.9)	1233.6 (279.1)	58.8 (37.8)	101.5 (15.1)	74.6 (32.7)	139.2 (41.5)	134.0 (111.2)	390.8 (166.9)
	**Psychologist**	44.4 (24.7)	9.8 (3.0)	5.5 (5.5)	4.7 (3.6)	544.3 (202.0)	526.1 (206.0)	4.4 (3.0)	32.8 (7.7)	5.8 (4.0)	12.3 (7.0)	57.2 (36.3)	90.1 (34.6)
	**Psychiatrist**	3.8 (1.6)	4.8 (1.3)	0.0 (0.0)	2.9 (2.3)	11.6 (8.6)	3.5 (3.5)	9.4 (6.4)	2.6 (0.8)	30.6 (30.6)	57.5 (45.5)	21.4 (21.4)	96.2 (51.9)
	**Physiotherapist**	206.0 (52.4)	118.7 (15.5)	178.8 (132.5)	105.5 (38.6)	1365.4 (427.2)	807.1 (250.2)	4.6 (4.6)	342.5 (85.5)	177.4 (127.4)	242.4 (78.0)	74.0 (33.9)	109.2 (50.9)
	**Respiratory specialist**	10.3 (3.7)	4.9 (0.8)	0.0 (0.0)	9.5 (3.7)	256.6 (66.6)	262.9 (52.3)	5.9 (5.9)	44.5 (27.4)	19.7 (9.7)	20.1 (10.7)	55.0 (19.5)	14.4 (3.5)
**1)Total Hospital outpatient services**		603.6 (98.2)	381.7 (29.0)	379.7 (153.6)	440.9 (87.7)	4459.1 (844.3)	3580.8 (655.3)	308.5 (68.2)	759.9 (99.1)	958.0 (262.9)	929.1 (184.5)	1697.8 (491.8)	1299.7 (276.3)
**2) Hospital inpatient services**		3363.0 (1265.4)	973.0 (204.9)	495.7 (296.5)	134.3 (50.6)	180.4 (55.1)	167.0 (49.5)	624.2 (496.0)	108.3 (29.0)	1264.5 (589.5)	91.5 (25.9)	966.8 (489.1)	382.7 (166.7)

**3) Community health and social services**	**GP**	4.9 (2.2)	6.3 (1.2)	32.3 (9.0)	51.6 (5.5)	441.3 (108.1)	268.6 (33.2)	79.2 (26.2)	22.0 (2.2)	119.9 (25.5)	116.2 (9.6)	60.1 (28.4)	77.8 (10.0)
	**Dietician**	0.4 (0.4)	1.5 (0.6)	5.8 (5.8)	2.4 (1.4)	7.1 (4.6)	0.9 (0.7)	3.0 (3.0)	2.0 (0.6)	56.0 (21.4)	14.2 (3.7)	71.0 (33.3)	18.7 (6.9)
	**Health visitor**	92.4 (92.4)	4.1 (3.2)	0.0 (0.0)	5.0 (3.7)	8.5 (7.8)	5.0 (1.9)	3.2 (3.2)	12.7 (6.8)	4.8 (3.3)	10.7 (3.5)	0.0 (0.0)	0.0 (0.0)
	**School Nurse**	0.0 (0.0)	0.0 (0.0)	0.0 (0.0)	12.1 (2.0)	3.9 (1.9)	4.5 (1.5)	0.0 (0.0)	3.0 (0.8)	19.1 (8.9)	32.7 (7.6)	0.0 (0.0)	0.0 (0.0)
	**Occupational therapist**	3.7 (3.7)	2.4 (1.4)	19.2 (19.2)	7.3 (5.5)	1120.2 (370.9)	469.6 (188.2)	77.7 (77.7)	64.8 (12.3)	114.9 (45.6)	226.8 (100.3)	208.6 (172.4)	310.6 (247.0)
	**Early childhood intervention**	91.9 (52.6)	23.1 (8.0)	0.0 (0.0)	1.7 (1.7)	361.0 (150.1)	605.9 (277.7)	771.4 (442.3)	168.8 (29.6)	6717.2 (4805.1)	3920.3 (2004.2)	237.0 (146.4)	120.0 (42.8)
	**Other costs**	0.0 (0.0)	0.0 (0.0)	28.1 (9.4)	15.3 (2.3)	114.8 (15.4)	84.1 (9.8)	21.9 (9.4)	11.8 (1.5)	26.0 (13.7)	3.6 (1.0)	323.0 (93.6)	186.6 (29.6)
**3) Total Community health and social services**		193.3 (105.2)	37.5 (9.4)	85.3 (24.0)	95.5 (9.2)	2056.8 (464.4)	1438.7 (432.3)	956.4 (532.6)	285.2 (40.9)	7057.9 (4835.3)	4324.7 (2037.9)	899.7 (350.4)	713.6 (256.6)
**4) Equipment costs[1]**		41.0 (20.0)	42.2 (19.8)	9.4 (7.2)	22.0 (9.8)	535.8 (207.7)	411.9 (210.8)	0.0 (0.0)	37.3 (8.7)	259.6 (248.1)	45.0 (34.4)	0.0 (0.0)	502.0 (446.6)
**5) Family expenses[2]**		690.3 (352.9)	243.4 (62.1)	194.4 (146.3)	8.8 (4.6)	2014.6 (727.7)	1392.4 (390.1)	81.2 (41.5)	164.1 (37.3)	96.8 (89.7)	132.9 (86.5)	123.8 (123.8)	3508.6 (2105.2)
**6) Valuation of time work off [3]**		1388.0 (732.0)	1058.4 (200.6)	87.5 (60.6)	839.0 (584.9)	1792.6 (949.2)	1826.7 (613.8)	1043.6 (875.4)	1413.4 (291.5)	149.6 (107.7)	1463.3 (440.0)	9492.2 (7670.3)	1183.9 (400.7)
**7) Total societal costs+**		6279.2 (1689.3)	2736.1 (336.9)	1252.0 (399.2)	1540.5 (654.0)	11039.3 (1927.4)	8817.4 (1576.7)	3013.8 (1230.3)	2768.3 (394.7)	9786.4 (5366.1)	6986.5 (2397.6)	13180.4 (8162.1)	7590.6 (2552.2)

CA denotes congenital anomalies.

[1] These costs include any special equipment related to infants such as wheelchairs, braces, hearing aids or shoes.

[2] These costs include any other expenses associated with the child's health.

[3] These costs include salaries related to time off work

[+] Sum of all the cost categories.

Appendix 8 Economic costs during the fifth year of life associated with congenital anomalies, by severity and by country

		Belgium	Denmark	Estonia	France	Germany	Italy	Netherlands	Poland	Portugal	UK	Sweden
**Hospital outpatient services (HOS)**	**Mild**	528.6 (296.7)	244.9 (244.9)	0.0 (.)	491.1 (213.0)	1071.5 (343.6)	577.7 (149.9)	0.0 (.)	5770.5 (1663.7)	0.0 (.)	1997.3 (841.4)	2206.3 (.)
	**Moderate**	0.0 (.)	14.8 (14.8)	3320.9 (2989.9)	1759.9 (643.9)	514.1 (218.8)	630.4 (145.9)	398.9 (162.2)	2913.5 (748.4)	267.0 (83.1)	821.7 (373.7)	0.0 (.)
	**Severe**	29.0 (.)	115.9 (42.2)	1709.9 (1441.7)	1337.4 (435.0)	1500.3 (271.3)	590.7 (237.1)	72.7 (.)	4187.6 (1473.6)	377.6 (122.2)	512.8 (173.1)	1901.8 (756.8)
**Hospital inpatient services (HIS)**	**Mild**	0.0 (.)	0.0 (.)	0.0 (.)	54.6 (37.8)	0.0 (.)	4547.3 (3134.0)	0.0 (.)	109.6 (61.0)	0.0 (.)	2662.2 (1687.6)	859.3 (.)
	**Moderate**	0.0 (.)	0.0 (.)	276.3 (276.3)	682.3 (357.5)	1309.5 (1309.5)	953.2 (520.4)	526.7 (313.9)	192.6 (110.6)	838.9 (795.5)	1109.2 (967.9)	0.0 (.)
	**Severe**	0.0 (.)	0.0 (.)	0.0 (.)	357.4 (357.4)	4293.6 (2575.1)	5863.6 (2937.1)	0.0 (.)	252.1 (118.7)	266.3 (168.4)	623.9 (509.4)	687.5 (280.7)
**Community health and social services (CHSS)**	**Mild**	112.5 (112.5)	61.4 (61.4)	0.0 (.)	62.3 (12.2)	907.1 (538.5)	315.7 (298.9)	0.0 (.)	2751.7 (939.0)	0.0 (.)	12973.3 (12684.3)	3034.0 (.)
	**Moderate**	0.0 (.)	52.8 (32.5)	331.4 (211.3)	52.3 (15.2)	520.4 (241.5)	221.5 (121.1)	88.5 (25.3)	891.4 (402.9)	443.6 (252.7)	355.4 (149.9)	0.0 (.)
	**Severe**	29.0 (.)	101.5 (101.5)	183.6 (106.6)	58.3 (17.5)	823.7 (311.3)	4.9 (3.8)	34.3 (.)	2190.0 (760.2)	1811.0 (1357.9)	13414.4 (13208.3)	794.6 (519.0)
**Equipment costs[1]**	**Mild**	31.3 (31.3)	0.0 (.)	0.0 (.)	3.5 (3.5)	0.0 (.)	67.9 (51.9)	0.0 (.)	483.0 (201.0)	0.0 (.)	1241.4 (1241.4)	0.0 (.)
	**Moderate**	0.0 (.)	1180.0 (1180.0)	95.9 (89.1)	27.6 (13.3)	8.0 (8.0)	26.1 (26.1)	10.0 (7.6)	260.0 (203.1)	0.0 (.)	23.6 (23.6)	0.0 (.)
	**Severe**	174.0 (.)	0.0 (.)	91.3 (91.3)	23.8 (13.3)	91.8 (51.4)	33.6 (19.4)	0.0 (.)	817.0 (548.8)	0.0 (.)	0.0 (.)	0.0 (.)
**Family expenses[2]**	**Mild**	0.0 (.)	0.0 (.)	0.0 (.)	32.8 (32.1)	0.0 (.)	271.0 (240.8)	0.0 (.)	1111.8 (451.1)	0.0 (.)	448.4 (448.4)	0.0 (.)
	**Moderate**	0.0 (.)	118.0 (118.0)	0.0 (.)	30.6 (21.4)	1.6 (1.6)	448.4 (274.0)	206.5 (155.2)	1779.4 (1268.1)	86.6 (53.3)	0.0 (.)	0.0 (.)
	**Severe**	0.0 (.)	0.0 (.)	557.1 (557.1)	125.7 (125.7)	455.8 (196.6)	1569.3 (1229.6)	0.0 (.)	3240.9 (1830.6)	72.2 (72.2)	22.1 (22.1)	0.0 (.)
**Valuation of time work off**	**Mild**	913.4 (913.4)	0.0 (.)	0.0 (.)	106.7 (71.2)	346.2 (230.9)	959.1 (598.9)	0.0 (.)	471.3 (262.8)	0.0 (.)	476.1 (476.1)	10170.2 (.)
	**Moderate**	0.0 (.)	0.0 (.)	664.2 (419.1)	366.1 (203.3)	910.0 (445.3)	1838.4 (1655.3)	92.9 (64.2)	5522.2 (3373.1)	1669.7 (1388.3)	0.0 (.)	0.0 (.)
	**Severe**	0.0 (.)	0.0 (.)	5165.8 (5165.8)	425.8 (235.2)	1481.8 (467.9)	1162.8 (425.1)	0.0 (.)	328.5 (175.6)	0.0 (.)	170.0 (170.0)	13763.7 (12271.0)

**Total societal cost**	**Mild**	1585.9 (760.6)	306.3 (306.3)	0.0 (.)	751.0 (268.6)	2324.9 (709.9)	6738.8 (3834.8)	0.0 (.)	10697.8 (2605.7)	0.0 (.)	19798.6 (17083.5)	16269.9 (.)
	**Moderate**	0.0 (.)	1365.6 (1195.4)	4688.6 (3143.6)	2918.8 (990.9)	3263.5 (1901.2)	4118.0 (1860.8)	1323.5 (418.1)	11559.1 (3253.1)	3305.9 (1835.2)	2310.0 (1414.3)	0.0 (.)
	**Severe**	232.0 (.)	217.4 (59.3)	7707.8 (4524.3)	2328.5 (786.4)	8647.1 (2764.6)	9224.9 (3485.2)	107.0 (	11016.1 (4208.	2527.1 (1388.8)	14743.3 (13233.7)	17147.5 (13061.4)

(continued)

		**Belgium**	**Denmark**	**Estonia**	**France**	**Germany**	**Italy**	**Netherlands**	**Poland**	**Portugal**	**UK**	**Sweden**
**Hospital outpatient services (HOS)**	**Mild**	528.6 (296.7)	244.9 (244.9)	0.0 (.)	491.1 (213.0)	1071.5 (343.6)	577.7 (149.9)	0.0 (.)	5770.5 (1663.7)	0.0 (.)	1997.3 (841.4)	2206.3 (.)
	**Moderate**	0.0 (.)	14.8 (14.8)	3320.9 (2989.9)	1759.9 (643.9)	514.1 (218.8)	630.4 (145.9)	398.9 (162.2)	2913.5 (748.4)	267.0 (83.1)	821.7 (373.7)	0.0 (.)
	**Severe**	29.0 (.)	115.9 (42.2)	1709.9 (1441.7)	1337.4 (435.0)	1500.3 (271.3)	590.7 (237.1)	72.7 (.)	4187.6 (1473.6)	377.6 (122.2)	512.8 (173.1)	1901.8 (756.8)
**Hospital inpatient services (HIS)**	**Mild**	0.0 (.)	0.0 (.)	0.0 (.)	54.6 (37.8)	0.0 (.)	4547.3 (3134.0)	0.0 (.)	109.6 (61.0)	0.0 (.)	2662.2 (1687.6)	859.3 (.)
	**Moderate**	0.0 (.)	0.0 (.)	276.3 (276.3)	682.3 (357.5)	1309.5 (1309.5)	953.2 (520.4)	526.7 (313.9)	192.6 (110.6)	838.9 (795.5)	1109.2 (967.9)	0.0 (.)
	**Severe**	0.0 (.)	0.0 (.)	0.0 (.)	357.4 (357.4)	4293.6 (2575.1)	5863.6 (2937.1)	0.0 (.)	252.1 (118.7)	266.3 (168.4)	623.9 (509.4)	687.5 (280.7)
**Community health and social services (CHSS)**	**Mild**	112.5 (112.5)	61.4 (61.4)	0.0 (.)	62.3 (12.2)	907.1 (538.5)	315.7 (298.9)	0.0 (.)	2751.7 (939.0)	0.0 (.)	12973.3 (12684.3)	3034.0 (.)
	**Moderate**	0.0 (.)	52.8 (32.5)	331.4 (211.3)	52.3 (15.2)	520.4 (241.5)	221.5 (121.1)	88.5 (25.3)	891.4 (402.9)	443.6 (252.7)	355.4 (149.9)	0.0 (.)
	**Severe**	29.0 (.)	101.5 (101.5)	183.6 (106.6)	58.3 (17.5)	823.7 (311.3)	4.9 (3.8)	34.3 (.)	2190.0 (760.2)	1811.0 (1357.9)	13414.4 (13208.3)	794.6 (519.0)
**Equipment costs¹**	**Mild**	31.3 (31.3)	0.0 (.)	0.0 (.)	3.5 (3.5)	0.0 (.)	67.9 (51.9)	0.0 (.)	483.0 (201.0)	0.0 (.)	1241.4 (1241.4)	0.0 (.)
	**Moderate**	0.0 (.)	1180.0 (1180.0)	95.9 (89.1)	27.6 (13.3)	8.0 (8.0)	26.1 (26.1)	10.0 (7.6)	260.0 (203.1)	0.0 (.)	23.6 (23.6)	0.0 (.)
	**Severe**	174.0 (.)	0.0 (.)	91.3 (91.3)	23.8 (13.3)	91.8 (51.4)	33.6 (19.4)	0.0 (.)	817.0 (548.8)	0.0 (.)	0.0 (.)	0.0 (.)
**Family expenses²**	**Mild**	0.0 (.)	0.0 (.)	0.0 (.)	32.8 (32.1)	0.0 (.)	271.0 (240.8)	0.0 (.)	1111.8 (451.1)	0.0 (.)	448.4 (448.4)	0.0 (.)
	**Moderate**	0.0 (.)	118.0 (118.0)	0.0 (.)	30.6 (21.4)	1.6 (1.6)	448.4 (274.0)	206.5 (155.2)	1779.4 (1268.1)	86.6 (53.3)	0.0 (.)	0.0 (.)
	**Severe**	0.0 (.)	0.0 (.)	557.1 (557.1)	125.7 (125.7)	455.8 (196.6)	1569.3 (1229.6)	0.0 (.)	3240.9 (1830.6)	72.2 (72.2)	22.1 (22.1)	0.0 (.)

**Valuation of time work off**	**Mild**	913.4 (913.4)	0.0 (.)	0.0 (.)	106.7 (71.2)	346.2 (230.9)	959.1 (598.9)	0.0 (.)	471.3 (262.8)	0.0 (.)	476.1 (476.1)	10170.2 (.)
	**Moderate**	0.0 (.)	0.0 (.)	664.2 (419.1)	366.1 (203.3)	910.0 (445.3)	1838.4 (1655.3)	92.9 (64.2)	5522.2 (3373.1)	1669.7 (1388.3)	0.0 (.)	0.0 (.)
	**Severe**	0.0 (.)	0.0 (.)	5165.8 (5165.8)	425.8 (235.2)	1481.8 (467.9)	1162.8 (425.1)	0.0 (.)	328.5 (175.6)	0.0 (.)	170.0 (170.0)	13763.7 (12271.0)
**Total societal cost**	**Mild**	1585.9 (760.6)	306.3 (306.3)	0.0 (.)	751.0 (268.6)	2324.9 (709.9)	6738.8 (3834.8)	0.0 (.)	10697.8 (2605.7)	0.0 (.)	19798.6 (17083.5)	16269.9 (.)
	**Moderate**	0.0 (.)	1365.6 (1195.4)	4688.6 (3143.6)	2918.8 (990.9)	3263.5 (1901.2)	4118.0 (1860.8)	1323.5 (418.1)	11559.1 (3253.1)	3305.9 (1835.2)	2310.0 (1414.3)	0.0 (.)
	**Severe**	232.0 (.)	217.4 (59.3)	7707.8 (4524.3)	2328.5 (786.4)	8647.1 (2764.6)	9224.9 (3485.2)	107.0 (	11016.1 (4208.	2527.1 (1388.8)	14743.3 (13233.7)	17147.5 (13061.4)

Appendix 9 Multi-level GLM regressions of factors associated with economic costs of very preterm children during the fifth year of life, by severity of congenital anomaly, mean (SE)

		Total health and social care services costs			Total societal costs		
		(1)	(2)	(3)	(4)	(5)	(6)
**Congenital anomaly (reference: no congenital anomaly)**	**Mild**	2260*	1814*	1542*	3218	2440	1948
		(901)	(788)	(731)	(637240)	(1380)	(1284)
	**Moderate**	1232*	782	728	2451	1674	1631
		(550)	(464)	(453)	(485470)	(1028)	(1026)
	**Severe**	4537***	4229***	3930**	8467	8011**	6908**
		(1371)	(1281)	(1238)	(1700000)	(2617)	(2413)
**Employment status (reference: Employed or other situation)**	**At least one parent unemployed**	459	332	378	831	533	631
		(277)	(257)	(266)	(164519)	(538)	(558)
**Mother's education (reference: High education ISCED 6-8)**	**Intermediate education ISCED 3-5**	216	217	177	204	209	116
		(152)	(147)	(150)	(40387)	(318)	(326)
	**Low education ISCED 0-2**	211	269	221	-11	147	74
		(211)	(211)	(212)	(2173)	(435)	(442)
**Country of birth for mothers (reference: native)**	**Non-native, European born**	-632*	-652**	-539*	-1481	-1478**	-1494**
		(247)	(238)	(263)	(293309)	(481)	(498)
	**Non-native, non-European born**	-838***	-881***	-820***	-1671	-1810***	-1691***
		(156)	(149)	(156)	(330925)	(324)	(339)
**Mother's age at childbirth (years) (reference: 25-34)**	**<25**	-104	-111	-144	-97	-136	-299
		(225)	(216)	(219)	(19108)	(454)	(452)
	**>34**	-187	-122	-117	-2	150	176
		(150)	(149)	(152)	(449)	(331)	(341)

**Parity (reference: multiparous)**	**Nulliparous**	48	109	286	331
		(141)	(142)	(299)	(305)
**Multiples (reference: singleton)**	**Twins**	-322*	-311*	-584	-580
		(145)	(147)	(312)	(318)
	**Triplets**	-260	-222	-302	-227
		(421)	(434)	(951)	(982)
**Gender (reference: Female)**	**Male**	775***	794***	1613***	1609***
		(132)	(135)	(285)	(291)
**SGA (reference:>10th centile)**	**<3rd centile**	314.0	254	555	503
		(217.1)	(217)	(445)	(449)
	**3 to 10**	284.2	170	725	580
		(171.4)	(171)	(374)	(377)
**Gestational age (weeks) (reference: 30-31)**	**<26**	3541***	1559***	8096***	3837***
		(618)	(411)	(1478)	(982)
	**26-27**	820***	440*	1608***	934*
		(195)	(187)	(414)	(398)
	**28-29**	407**	283	677*	460
		(138)	(147)	(283)	(302)
**BPD (reference: no BPD)**	**Yes**		772***		1160*
			(224)		(464)
**Severe non-respiratory morbidity (reference: no severe non-respiratory morbidity)**	**Yes**		2501***		5376***
			(464)		(1029)

1: Model (1) and (4) : (i) Socioeconomic characteristics
2: Model (2) and (5) : (i) Socioeconomic characteristics + (ii) Clinical characteristics
3: Model (3) and (6) : (i) Socioeconomic characteristics + (ii) Clinical characteristics + (iii) Complications of preterm birth

ISCED: International Standard Classification of Education

Standard errors in parentheses * $p<0.05$; ** $p<0.01$; *** $p<0.001$

Appendix 10 The resource use questionnaire used in this study

☐☐☐☐

SHIPS ID

(for office use)

Questionnaire

Your premature child's health, care and development at 5 years of age

Name of child: ………………………………………………………………………………………

Date of birth: [PRINTED]

In this form we ask you to answer some questions about your child and your family, now that he or she is five years of age. This information will tell us about your child's current health and development, and what kind of health care he or she has used over

the last year. Your answers will help us find out how best to support the learning and development children who were born prematurely.
All children develop differently and have different strengths and needs, so you may feel that some of the questions do not apply to your child. However, please answer all the questions to the best of your ability, even if you feel they do not apply to your child, as all the information gained is important for the study.

Please start by filing in

Today's date: D D | M M | 2 0 Y Y

SHIPS ID (for office use) ☐☐☐☐

Childcare

In this section we ask some questions about who cares for your child and whether your child attends [standard educational program in country at this age]. We need this background information to tell us about the kinds of everyday experiences your child has.

1.1 **What is your relationship to the child?**

☐ Mother ☐ Father ☐ Other relative or family member ☐ Other caregiver

1.2 **Please tell us who your child lives with.** Please tick only one box.

☐ Both parents ☐ Mother only

☐ Father only ☐ Alternates between mother and father

☐ Other relative or family member ☐ With someone else, please specify:

………………………………………………………………………………………

1.3a **Is your child currently in preschool (for example, nursery or kindergarten), school or other educational programme?**

☐ Yes ☐ No

If your child is not in any educational programme, please go to **question 2.1.**

1.3b **If you answered yes, please specify the type of preschool, school or other educational programme your child currently attends**. If your child attends more than one, please tell us about each one:

**Type of educational programme**<br>For example nursery, kindergarten, school, special education or regular education.	**Age when your child started full or part-time?** Years	Months	**Does your child attend** Full-time	Part-time
			☐	☐

1.3c **Do you feel your child *needs* special educational support or services?** ☐ Yes ☐ No

1.3d **Does your child *have* special educational support or services?** ☐ Yes ☐ No

1.3e **If yes, please describe what type of support/service your child has:**

....

Your child's health and development

In this section we ask some questions about your child's health, learning and development. This will help us understand more about the needs of children born prematurely, and the kinds of concerns parents of prematurely born children may have as their child grows older.

2.1 **How would you rate your child's overall health?**

☐ Excellent ☐ Good ☐ Fair ☐ Poor

2.2 **How would you rate your child's overall development?**

☐ Average ☐ In advance ☐ Some delay ☐ Very delayed

Cms ***OR*** Ft ins

2.3a **What is your child's height?** [ ][ ][ ] [ ] [ ][ ]

2.3b **Date when recorded:** D D / M M / 2 0 Y Y

2.3c **Recorded by:**

☐ Health care professional ☐ Parent or caregiver

Kg ***OR*** lbs oz

2.4a **What is your child's weight?** [ ][ ] , [ ][ ] [ ][ ] . [ ][ ]

2.4b **Date when recorded:** D D / M M / 2 0 Y Y

2.4c **Recorded by:**

☐ Health care professional ☐ Parent or caregiver

2.5a **Does your child have any difficulties with his or her vision?** Please tick only one box.

- ☐ No difficulties with vision
- ☐ Needs to wear glasses but sees well when wearing them
- ☐ Has difficulties seeing, even wearing glasses
- ☐ Is blind, or able to see light only

2.5b **If your child has difficulties with his or her vision, please specify the age at which the difficulties were identified:** ☐ ☐☐ **Years Months**

2.6a **Does your child have any difficulties hearing?** Please tick only one box.

- ☐ No difficulties hearing
- ☐ Some difficulties hearing but does not require hearing aids or cochlear implants
- ☐ Needs hearing aids or cochlear implants, but hears well with them
- ☐ Has difficulty hearing, even with hearing aids or cochlear implants ☐ My child is deaf

2.6b **If your child has difficulties hearing, please specify the age at which the difficulties were identified:** ☐ ☐☐ **Years Months**

2.7 **Does your child have any difficulties using his or her hands and fingers to handle small objects, such as buttoning buttons?** Please tick only one box.

- ☐ No difficulties using hands and fingers

☐ Some difficulties, but is able to handle small objects without help

☐ Needs help from an adult to handle small objects

☐ Is unable to handle small objects at all

2.8 **Does your child have any difficulties walking?** Please tick only one box.

☐ No difficulties walking

☐ Can walk alone but is unsteady

☐ Can only walk with help from an adult or walking aid

☐ Unable to walk even with help from an adult or walking aid

2.9a **Has your child been given a diagnosis of cerebral palsy by a doctor or other health professional?** ☐ Yes ☐ No

2.9b **If yes, how old was your child when he or she was diagnosed with cerebral palsy?**

Years [ ] **Months** [ ][ ]

2.10 **Has your child been given a diagnosis of any of the following conditions by a doctor or other health professional?** Please give an answer for each diagnosis.

**Condition**	**Yes**	**No**	**Don't know**	**Age when diagnosed** Years	Months
**Asthma**	☐☐ ☐☐ ☐☐ ☐☐☐ ☐				
ADHD (Attention Deficit/Hyperactivity Disorder)	☐	☐	☐		

Autism or Autism Spectrum Disorder	☐	☐	☐
Language or speech delay	☐	☐	☐
**Epilepsy**			
Developmental delay	☐	☐	☐

Age when diagnosed

If your child has been given any other diagnosis, please specify below

	Years	Months

2.11 **Apart from any health concerns and diagnosis already mentioned, do you have other concerns about the health and development of your child?** Please tick a box for each of the concerns you currently may have regarding your child's… If you have no concerns, leave blank.

☐ Speech ☐ Hearing ☐ Vision

☐ Weight ☐ Height ☐ Sleep

☐ Appetite ☐ Anxiety Such as fears, worries or phobias ☐ Learning

- ☐ **Breathing** — Out of breath, difficulties breathing
- ☐ **Social interactions** — For example difficulties talking to or playing with other children
- ☐ **Eating** — For example chewing, swallowing
- ☐ **Behavior** — For example difficulties paying attention, sitting still, following instructions or not thinking things out before acting
- ☐ **Movement and coordination** — For example clumsiness, or having difficulties catching a ball or dressing him or herself

If you have any other concerns not mentioned above, please describe below what worries you:

....

....

....

....

....

Your child’s communication and problem solving

Please answer the following questions together with your child. Some of the questions involve asking your child to have a go at an activity or task with you. You may wish to come back to this part of the questionnaire later, when it is a good time for you and your child to try the activities.

These questions are about activities children may do. Your child may have already done some of the activities described here, and there may be some your child has not begun doing yet. For each item, please tick the box that indicates whether your child is doing the activity regularly, sometimes, or not yet.

Important Points to Remember:

- Try each activity with your child before marking a response.
- Make completing this questionnaire a game that is fun for you and your child.
- Make sure your child is rested and fed.

COMMUNICATION

3.1 **Without your giving help by pointing or repeating directions,** Yes Sometimes Not Yet **does your child follow three directions that are unrelated to one** ☐ ☐ ☐ **another?**

Give all three directions before your child starts. For example, you may ask your child to:

"Clap your hands, walk to the door, and sit down." or
"Give me the pen, open the book, and stand up."

Yes Sometimes Not Yet

☐ ☐ ☐

3.2 **Does your child use four- and five-word sentences?**

For example, does your child say, "I want the car"?

Please write an example: ………

3.3. **When talking about something that already happened, does your child use words that end in "ed," such as "walked", "jumped" or "played"?** Yes ☐ Sometimes ☐ Not Yet ☐

Ask your child questions, such as:

"How did you get to the store?" ("We walked.")
"What did you do at your friend's house?" ("We played.")

Please write an example: ………

From Ages & Stages Questionnaires®, Third Edition (ASQ-3™), Squires & Bricker, Pilot Version 60m +.
© 2009-2016 Paul H. Brookes Publishing Co., Inc. Used with permission.

3.4 **Does your child use comparison words, such as heavier, stronger, or shorter?** Yes Sometimes Not Yet

☐ ☐ ☐

Ask your child questions, such as:

“A car is big, but a bus is _____” (bigger); “A cat is heavy, but a man is _____” (heavier); “A TV is small, but a book is _____” (smaller).

Please write an example: ……

3.5 **Does your child answer the following questions:** Yes Sometimes Not Yet

☐ ☐ ☐

Mark “sometimes” if your child answers only one question

“What do you do when you are hungry?”
Acceptable answers include: “get food”, “eat”, “ask for something to eat”, and “have a snack”.

Please write your child’s response: ……………………………………………………………………………………

“What do you do when you are tired?”
Acceptable answers include: “take a nap”, “rest”, “go to sleep”, “go to bed”, “lie down”, and “sit down”.

Please write your child’s response: ………………………………………………………………………………………

Yes Sometimes Not Yet

3.6 **Does your child repeat the sentences shown below back to you,**

without any mistakes? ☐ ☐ ☐

Read the sentences one at a time. You may repeat each sentence one time.

Mark "yes" if your child repeats both sentences without mistakes or "sometimes" if your child repeats one sentence without mistakes.

"Jane hides her shoes for Maria to find."

"Al read the blue book under his bed."

Yes Sometimes Not Yet

3.7 **After hearing new words, does your child try to use them in conversation?** ☐ ☐ ☐

Yes Sometimes Not Yet

3.8 **Does your child use at least 5 words to show where things are?**

For example, does she use words such as "above", "across", "around", "between", "below", "near", "over", and "through"?

From Ages & Stages Questionnaires®, Third Edition (ASQ-3™), Squires & Bricker, Pilot Version 60m +.
© 2009-2016 Paul H. Brookes Publishing Co., Inc. Used with permission.

Yes Sometimes Not Yet

3.9 **Does your child use words that connect other words such as** ☐ ☐ ☐ **"and", "but", "because", "if", and "or"?**

For example, your child says "We could play or take a nap".

PROBLEM SOLVING

Yes Sometimes Not Yet

☐ ☐ ☐

3.10 **When asked, "Which circle is the smallest?" does your child point to the smallest circle?**

Ask this question without providing help by pointing, gesturing, or looking at the smallest circle.

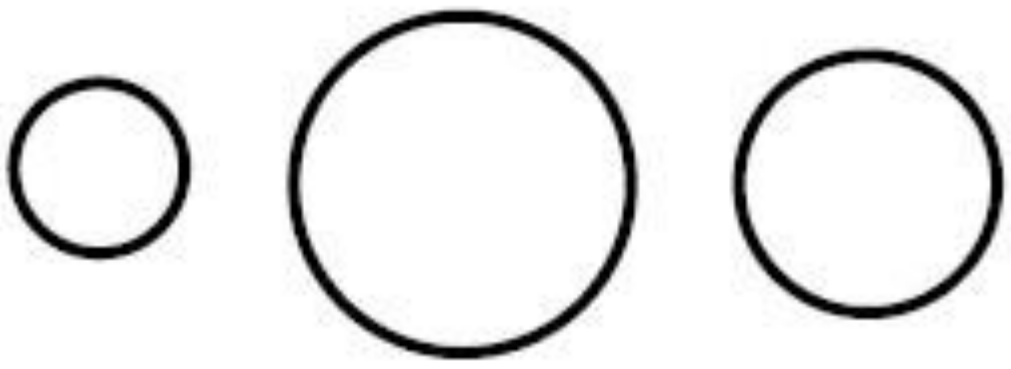

3.11 **When shown objects and asked, "What color is this?" does** Yes Sometimes Not Yet **your child name five different colors like red, blue, yellow, orange,**

☐ ☐ ☐ **blac**

white, or pink?

Mark "yes" only if your child answers the question correctly using five colors.

3.12 **Does your child count up to 15 without making mistakes?**

Yes Sometimes Not Yet

☐ ☐ ☐

If so, mark "yes." If your child counts to 12 without making mistakes, mark "sometimes."

Yes Sometimes Not Yet

☐ ☐ ☐

3.13 **Does your child finish the following sentences using a word that means the opposite of the word that is *italicized*?**

For example: "A rock is *hard*, and a pillow is *soft*."

Please write your child's responses below:

A cow is *big*, and a mouse is ________________________________.

Ice is *cold*, and fire is ____.

We see stars at *night*, and we see the sun during the _____________.

When I throw the ball *up*, it comes ________________________.

Mark "yes" if she finishes three of four sentences correctly. Mark "sometimes" if she finishes two of four sentences correctly.

From Ages & Stages Questionnaires®, Third Edition (ASQ-3™), Squires & Bricker, Pilot Version 60m +.
© 2009-2016 Paul H. Brookes Publishing Co., Inc. Used with permission.

3.14 **Does your child know the names of numbers?**

Yes Sometimes Not Yet

☐ ☐ ☐

Mark "yes" if he identifies the three numbers (3, 1, 2) below.
Mark "sometimes" if he identifies two numbers.

3 1 2

3.15 **Does your child name at least four letters in her name?**

Yes Sometimes Not Yet
☐ ☐ ☐

Point to the letters and ask, "What letter is this?" Point to the letters out of order.

3.16 **Can your child show you her left hand or her left foot?**

Yes Sometimes Not Yet
☐ ☐ ☐

3.17 **Can your child tell you what shape comes next in the pattern?**

Yes Sometimes Not Yet
☐ ☐ ☐

For example you may ask your child, "What comes after car?"

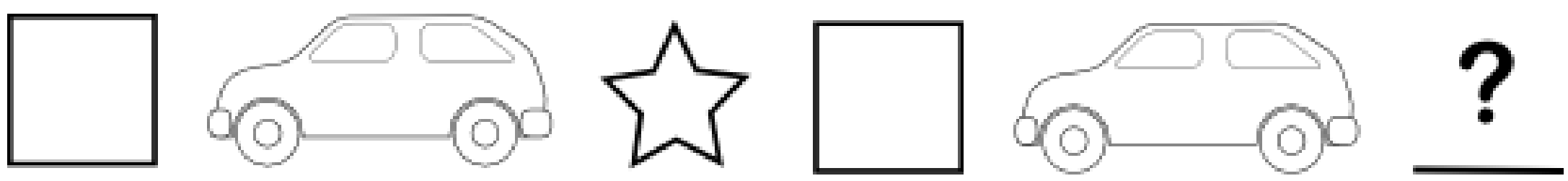

3.18 **Can your child count backwards starting at 10 and ending at 1?**

Mark "sometimes" if she counts at least 5 numbers backwards in order.

Yes Sometimes Not Yet
☐ ☐ ☐

From Ages & Stages Questionnaires®, Third Edition (ASQ-3™), Squires & Bricker, Pilot Version 60m +. © 2009-2016 Paul H. Brookes Publishing Co., Inc. Used with permission.

Your child's behavior and emotions

In this section we ask about your child's behavior and emotions. For each item, please mark the box for Not True, Somewhat True or Certainly True. It would help us if you answered all items as best you can even if you are not absolutely certain, or the question seems daft!

4.1 Please give your answers on the basis of the child's behavior over the last **six months**

	Not true	Somewhat true	Certainly true
Considerate of other people's feelings	☐	☐	☐
Restless, overactive, cannot stay still for long	☐	☐	☐
Often complains of headaches, stomach-aches or sickness	☐	☐	☐
Shares readily with other children (treats, toys, pencils etc.)	☐	☐	☐
Often has temper tantrums or hot tempers	☐	☐	☐
Rather solitary, tends to play alone	☐	☐	☐
Generally obedient, usually does what adults request	☐	☐	☐
Many worries, often seems worried	☐	☐	☐
Helpful if someone is hurt, upset or feeling ill	☐	☐	☐
Constantly fidgeting or squirming	☐	☐	☐

Has at least one good friend	☐	☐	☐
Often fights with other children or bullies them	☐	☐	☐
Often unhappy, down-hearted or tearful	☐	☐	☐
Generally liked by other children	☐	☐	☐
Easily distracted, concentration wanders	☐	☐	☐
Nervous or clingy in new situations, easily loses confidence	☐	☐	☐
Kind to younger children	☐	☐	☐
Often lies or cheats	☐	☐	☐
Picked on or bullied by other children	☐	☐	☐
Often volunteers to help others (parents, teachers, other children)	☐	☐	☐
Thinks things out before acting	☐	☐	☐
Steals from home, school or elsewhere	☐	☐	☐
Gets on better with adults than with other children	☐	☐	☐
Many fears, easily scared	☐	☐	☐
Sees tasks through to the end, good attention span	☐	☐	☐

Strengths and Difficulties Questionnaire © Robert Goodman, 2005

Below is a list of things that might be a problem for your child. Please tell us how much of a problem each one has been for your child during the past ONE month. There are no right or wrong answers. Some questions may not apply to your child. For example, your child might not yet be in school or preschool. In this case, just leave the question blank.

4.2 **In the past <u>one month</u>, how much of a problem has your child had with…**

	Never	Almost never	Some times	Often	Almost always
PHYSICAL FUNCTIONING (problems with…)					
1. Walking 100 metres	☐	☐	☐	☐	☐
2. Running	☐	☐	☐	☐	☐
3. Participating in sports activity or exercise	☐	☐	☐	☐	☐
4. Lifting something heavy	☐	☐	☐	☐	☐
5. Taking a bath or shower by him or herself	☐	☐	☐	☐	☐
6. Doing chores, like picking up his or her toys	☐	☐	☐	☐	☐
7. Having aches or pains	☐	☐	☐	☐	☐
8. Low energy level	☐	☐	☐	☐	☐
EMOTIONAL FUNCTIONING (problems with…)					
1. Feeling afraid or scared	☐	☐	☐	☐	☐
2. Feeling sad	☐	☐	☐	☐	☐
3. Feeling angry	☐	☐	☐	☐	☐
4. Trouble sleeping	☐	☐	☐	☐	☐
5. Worrying about what will happen to him or her	☐	☐	☐	☐	☐
SOCIAL FUNCTIONING (problems with…)					
1. Getting along with other children	☐	☐	☐	☐	☐
2. Other children not wanting to be his or her friend	☐	☐	☐	☐	☐
3. Getting teased by other children	☐	☐	☐	☐	☐
4. Not able to do things that other children his or her age can do	☐	☐	☐	☐	☐

5. Keeping up when playing with other children	☐	☐	☐	☐	☐
SCHOOL FUNCTIONING (problems with…)					
1. Paying attention in class	☐	☐	☐	☐	☐
2. Forgetting things	☐	☐	☐	☐	☐
3. Keeping up with school activities	☐	☐	☐	☐	☐
4. Missing school because of not feeling well	☐	☐	☐	☐	☐
5. Missing school to go to the doctor or hospital	☐	☐	☐	☐	☐

PedsQL™ Copyright © 1998 JW Varni, Ph.D. All rights reserved.

Use of health care services during the past year

The following questions ask about the health care services your child has used during the last year. Using your answers will help us find out what kind of care children born prematurely usually receive and how much care they need at the age of five years.

5.1 **How many times has your child seen these professionals or used these services over the __last year?__** This includes appointments both at the health care office and at home.

Please provide the **__total number__** of visits for each care provider. If you do not remember the exact number of visits, write down your best guess. Write "0" if your child has not used the service in the last year.

	**Number** of visits
A child doctor (paediatrician) (because of illness, not routine checks or vaccination visits)	
A family doctor or general practitioner	

(because of illness, not routine checks or vaccination visits)		
Hospital Accident and Emergency department (A&E)		
Neurologist (brain specialist)		
An ear specialist or ENT (Ear, Nose and Throat specialist)		
An eye specialist (ophthalmologist)		
Speech / language therapist		
Psychologist		
Psychiatrist (because of anxiety, behavioral or developmental problems)		
Physiotherapist or motor development therapist		
Respiratory or asthma specialist		
Dietician		
Health visitor		
School nurse		
Occupational therapist		
Early intervention services (services that help children with disabilities or developmental delays to develop their skills and enhance their capacities)		
If other care provider, please specify:	**Number** of visits	

5.2a **Has your child had to stay in hospital overnight over the <u>last year?</u>** ☐ Yes ☐ No If you answered no, go to **question 5.3**

5.2b **If yes, please give information below for each time your child stayed in hospital**

**Type of ward** For example general paediatric ward or surgical ward	**Number of nights**		**Was surgery done?** No	**Was surgery done?** Yes	**If yes, please specify type of surgery**
			☐	☐	
			☐	☐	
			☐	☐	
			☐	☐	

5.3 **Does your child have routine check-ups for children who were born prematurely [Description of regional service]?**

☐ **No, never** had such check-ups.

☐ **No, not anymore.** Please specify age at last check-up: Years [ ] Months [ ][ ]

☐ **Yes, still has check-ups.** Please specify place or health care professional:

☐ Neonatal unit where he or she was born

☐ Other place or health care professional:…………………………………………………………………….

5.4 **How would you describe the health care your child has received related to his or her preterm birth, after being discharged from the neonatal unit?**

☐ Excellent ☐ Good ☐ Fair ☐ Poor ☐ Has not received any health care related to very preterm birth

5.5 **In your experience, how could the health care for very preterm children be improved?**

Skip this question if you have twins or triplets and already answered this question

....

....

....

....

....

Health care expenses during the last year

In this section we would like to know if you have had any additional expenses because you are caring for your premature child. This is important, because we want to know how this health care impacts on families financially.

6.1 **If your child has needed any special equipment related to his or her health over the __last year__ (for example wheelchair, brace, hearing aids or shoes) please tell us about:** If he or she did not receive any special equipment, leave blank.

**The type of equipment**	Who paid or reimbursed you for it<br>If partially paid for, specify both payers.	Cost to you over the last year<br>**(if applicable)**

6.2 **If you/your family had any other costs as a result of your child's health over the __last year__ please tell us about:** These costs could be for example for making changes to your home, medications, extra travel or child care as a result of visits to the doctor. If you had no other costs, leave blank.

**Reason for the cost**	Who paid or reimbursed you for it<br>If partially paid for, specify both payers.	Cost to you over the last year<br>**(if applicable)**

6.3a **Has your child's health meant that you, or another family member or caregiver (for example your partner), has had to take time off work and/or been unable to work over the __last year?__** You can tick several answers. Tick "no" if not in employment, were able to work from home or had flexible hours.

Yes, I took time off or   Yes, another family member or caregiver

☐ No ☐ was unable to work ☐ took time off or was unable to work

6.3b **If yes, how many days were taken off work as a result of your child's health in the last year?** Leave blank if you were able to work from home or had flexible hours.

For other family members:

For me: days   If several people, fill in the total days amount of days.

6.3c **If time was taken off work, was this time compensated for?**
For example by social security, an insurance or your employer. You can tick several boxes.

☐ No ☐ Yes, for me ☐ Yes, for another family member or caregiver

6.4a **During the <u>past year</u>, have you had difficulties that have prevented you** ☐ Yes ☐ No **from taking your child to health care services, or making special purchases for your child (such as buying medicines or glasses)?**

6.4b **If you answered yes, what were the difficulties?** You can tick several alternatives.

☐ Lack of money (for example cannot afford transport, consultation or equipment)

☐ Transportation difficulties (for example long distance or no transport)

- ☐ Difficulty getting child care for your other children
- ☐ Difficulty getting time off from work
- ☐ Lack of time (other commitments than work)
- ☐ Waiting times were too long
- ☐ Did not know which health care professional to consult

If other difficulties, please specify them below

Parental questions

These last questions concern your own health and wellbeing. Skip these questions if you have twins or triplets and already answered this part.

7.1 **What is your current family situation?**

☐ Married ☐ Single ☐ Cohabiting ☐ Other

7.2 **What language do you speak at home**

☐☐ [Main language of country] only ☐ Only other language(s) than [Main language of country] ☐ Both [Main language of country] and other language(s) ☐

7.3 **What is your country of birth?** ....

7.4a **How many other children are living in your household?** ☐☐ children under 18 years

Excluding the child participating in this study.

7.4b **How old are these children?** Ages: If no other children, leave blank.

7.5a **Please tick one box each for what best describes your and your partner's employment status.** If you do not live with a partner, skip filling in the "partner" column.

	Me	Partner
Full-time employed (including self-employment)	☐	☐
Part-time employed (including self-employment)	☐	☐
Unemployed	☐	☐
Student or apprentice	☐	☐
Parental leave	☐	☐
Home parent	☐	☐
Other	☐	☐
**If other, please specify**		
	☐	☐
	☐	☐

7.5b **If you are employed, how many hours per week do you work?** If not employed, leave blank. hours per week ☐

7.6 **Please tick <u>one</u> box each for what best describes your and your partner's highest educational level.** If you do not live with a partner, skip filling in the "partner" column.

	**Me**	**Partner**
Early childhood education	☐	☐
Primary	☐	☐
Lower secondary	☐	☐
Upper secondary	☐	☐
Post-secondary non-tertiary (pre-university courses)	☐	☐
Short cycle tertiary (vocational programmes)	☐	☐
Bachelor degree or equivalent	☐	☐
Master degree or equivalent	☐	☐
Doctoral degree or equivalent	☐	☐
Not elsewhere classified or uncertain	☐	☐
If not elsewhere classified or uncertain, please describe below		
	☐	☐
	☐	☐

These questions are about how you feel and how things have been with you <u>during the past 4 weeks</u>. For each question, please give the one answer that comes closest to the way you have been feeling.

7.7 **How much of the time, during the past <u>four weeks</u>...**

	All of the time	Most of the time	Some of the time	A little of the time	None of the time
have you been a very nervous person?	☐	☐	☐	☐	☐
felt calm and peaceful?	☐	☐	☐	☐	☐
felt downhearted and blue?	☐	☐	☐	☐	☐
been a happy person?	☐	☐	☐	☐	☐
felt so down in the dumps thatcould cheer you up? nothing	☐	☐	☐	☐	☐

These questions are derived from the 36-Item Health Survey, developed at RAND as part of the Medical Outcomes Study

7.8 **Are you part of a parent organization (for example [*regional organization*]) or a network (for example [*regional network*]) of parents of preterm children?**

☐ No, never ☐ No, only previously (but not anymore) ☐ Yes, currently

7.9 **Feel free to tell us if there is anything else you would like to add regarding your child's health and health care:**

....

……

……

……

……

……

Thank you very much for your time

ANNEX: OPTIONAL SECTIONS TO INCLUDE

Questionnaire

Your premature child's health, care and development at 5 years of age

Name of child: ……………………………………………………………………………………………

Date of birth: [PRINTED]

In this form we ask you to answer some questions about your child and your family, now that he or she is five years of age. This information will tell us about your child's current health and development, and what kind of health care he or she has used over the last year. Your answers will help us find out how best to support the learning and development children who were born prematurely.

All children develop differently and have different strengths and needs, so you may feel that some of the questions do not apply to your child. However, please answer all the questions to the best of your ability, even if you feel they do not apply to your child, as all the information gained is important for the study.

Mark the answer of your choice with an X: ☒ Yes ☐ No

If you wish to change your answer, cross out the first answer completely and mark the correct box with an X: ■ Yes ☒ No

Sometimes we ask you to write us a text answer, or to give us a number. Please answer in the space provided, in block letters or clear numbers.

Try to answer as accurately as possible. For example, if the answer is "two and a half years", write 2 years 6 months.

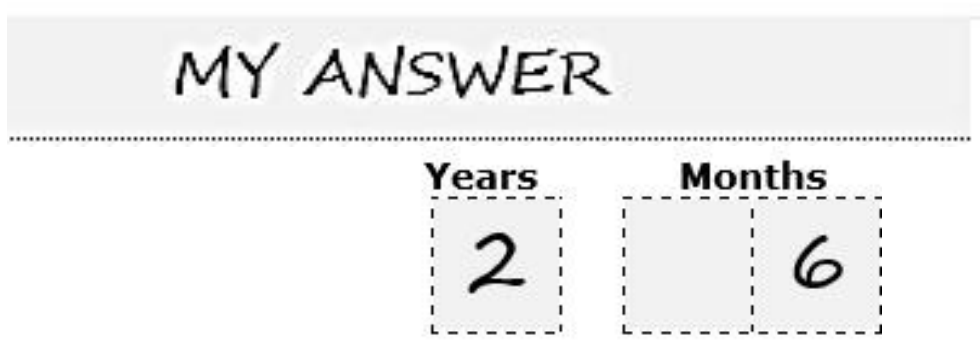

If you have any questions or would like any more information, please contact us on [local *number and email here*]. We are happy to answer any questions you have.

Please start by filing in

Today's date: D D | M M | 2 0 Y Y

Health care expenses during the last year

In this section we ask you about expenses you might have had as a result of the caring for your premature child. This is important, because we want to know how this health care impacts on families financially.

1. Are your child's health care expenses currently covered by a general health insurance, social security, a private insurance or increased child benefits [National healthcare system]? These expenses could be for example costs for medicines, purchasing equipment (hearing aid, shoes etc.), special need sessions or transportation. Please tick only one box.

☐ **No** costs)(only out of pocket ☐ **Yes, partially** (some out of pocket costs) ☐ **Yes, fully**pocket costs) (no out of

2a. Over the <u>last year</u>, have you been <u>refused reimbursement</u> by your health insurance provider for a request on costs for the care of your child?
For example costs for medicines, purchasing equipment (hearing aid, shoes etc.), for special needs sessions or transportation. Please tick only one box.

☐ **No –** because reimbursement cannot be requested for these costs

☐ **No –** because no request has been made

☐ **No -** request was accepted

☐ **Yes -** at least one refusal of a request made

2b. If refused, please specify which cost and why…………………………………………………………………

………

References for Appendix 3

1. Fund, C.H.I. *Logopedie*. 2017 [cited 2017 1 sep]; Available from: https://www.cm.be/diensten-en-voordelen/ziekte-en-behandeling/terugbetalingen-behandelingen/logopedisten.
2. Oostenbrink, J., Rutten-van Mölken, M., and Opdenoordt, T., *The treatment of newly diagnosed patients with glaucoma or with ocular hypertension in the Netherlands: an observational study of costs and initial treatment success based on retrospective chart review.* Documenta ophthalmologica, 1999. **98**(3): p. 285.
3. Jerusalem, G., et al., *Patterns of resource utilization and cost for postmenopausal women with hormone-receptor–positive, human epidermal growth factor receptor-2–negative advanced breast cancer in Europe.* BMC cancer, 2015. **15**(1): p. 787.
4. Kobelt, G., *Costs and quality of life for patients with multiple sclerosis in Belgium.* The European Journal of Health Economics, 2006. **7**(2): p. 24-33.
5. Jit, M., et al., *The cost-effectiveness of rotavirus vaccination: Comparative analyses for five European countries and transferability in Europe.* Vaccine, 2009. **27**(44): p. 6121-6128.
6. Kok, L., Lammers, M., Tempelman, C., and Bénard, H., *Remuneration of medical specialists: an international comparison*. 2012: SEO Economic Research.
7. Ankjær-Jensen, A. and Johansen, J.T., *The costs of seven case-vignettes in the Danish health sector.* DSI Danish Institute for Health Services Research, 2005.
8. Pohju, A., et al., *Adding a Dietitian to a Danish Liaison-Team after discharge of geriatric patients at nutritional risk may save health care costs.* Journal of Aging Science, 2016. **4**(159): p. 2.
9. Kocevar, V.S., et al., *Variations in pediatric asthma hospitalization rates and costs between and within Nordic countries.* Chest, 2004. **125**(5): p. 1680-1684A.
10. Toome, L., et al., *Väga enneaegsete vastsü ndinute ravitulemused ja ravikulud Eestis.* Tartu Ülikool, tervishoiu instituut, 2015.
11. Leal, J., et al., *Economic burden of cardiovascular diseases in the enlarged European Union.* European heart journal, 2006. **27**(13): p. 1610-1619.
12. Rapp, T., et al., *Exploring the relationship between Alzheimer's disease severity and longitudinal costs.* Value in Health, 2012. **15**(3): p. 412-419.
13. Bloudek, L., et al., *Cost of healthcare for patients with migraine in five European countries: results from the International Burden of Migraine Study (IBMS).* The journal of headache pain, 2012. **13**(5): p. 361.
14. Sarlon, E., et al., *A prospective study of health care resource utilisation and selected costs of schizophrenia in France.* BMC health services research, 2012. **12**(1): p. 269.
15. Lafuma, A. and Berdeaux, G., *Modelling lifetime cost consequences of ReSTOR® in cataract surgery in four European countries.* BMC ophthalmology, 2008. **8**(1): p. 12.
16. Doz, M., et al., *The association between asthma control, health care costs, and quality of life in France and Spain.* BMC pulmonary medicine, 2013. **13**(1): p. 15.
17. Sany, J., et al., *Medico-economic evaluation of infliximab in rheumatoid arthritis—prospective French study of a cohort of 635 patients monitored for two years.* Rheumatology, 2009. **48**(10): p. 1236-1241.

18. Gensichen, J., et al., *Cost-effectiveness of depression case management in small practices.* The British Journal of Psychiatry, 2013. **202**(6): p. 441-446.
19. Lorenz, K., et al., *Direct cost and predictive factors for treatment in patients with ocular hypertension or early, moderate and advanced primary open-angle glaucoma: the CoGIS study in Germany.* Graefe's Archive for Clinical Experimental Ophthalmology, 2013. **251**(8): p. 2019-2028.
20. Konnopka, A., et al., *Association of costs with somatic symptom severity in patients with medically unexplained symptoms.* Journal of psychosomatic research, 2013. **75**(4): p. 370-375.
21. Berg, J. and Lindgren, P., *Economic evaluation of FENO measurement in diagnosis and 1-year management of asthma in Germany.* Respiratory medicine, 2008. **102**(2): p. 219-231.
22. Riechmann, J., et al., *Costs of epilepsy and cost-driving factors in children, adolescents, and their caregivers in Germany.* Epilepsia, 2015. **56**(9): p. 1388-1397.
23. Italian Ministry of Health. *Nomenclatore dell'assistenza specialistica ambulatoriale*. 2013 [cited 2017 1 sep]; Available from: http://www.salute.gov.it/portale/temi/p2_6.jsp?lingua=italiano&id=1767&area=programmazioneSanitariaLea&menu=lea.
24. Vedovetto, A., Soriani, N., Merlo, E., and Gregori, D., *The burden of inappropriate emergency department pediatric visits: why Italy needs an urgent reform.* Health services research, 2014. **49**(4): p. 1290-1305.
25. Giaquinto, C., et al., *Costi della gastroenterite da rotavirus acquisita in comunità in età pediatrica a Padova in Italia.* PharmacoEconomics Italian Research Articles, 2007. **9**(2): p. 103-111.
26. Bolier, L., et al., *Cost-effectiveness of online positive psychology: Randomized controlled trial.* The Journal of Positive Psychology, 2014. **9**(5): p. 460-471.
27. Tan, S.S., Bouwmans, C.A., Rutten, F.F., and Hakkaart-van Roijen, L., *Update of the Dutch manual for costing in economic evaluations.* International journal of technology assessment in health care, 2012. **28**(2): p. 152-158.
28. De Bruijn, C., et al., *Cost-effectiveness of an education and activation program for patients with acute and subacute shoulder complaints compared to usual care.* International journal of technology assessment in health care, 2007. **23**(1): p. 80-88.
29. Bosmans, J.E. and Adriaanse, M.C., *Outpatient costs in pharmaceutically treated diabetes patients with and without a diagnosis of depression in a Dutch primary care setting.* BMC health services research, 2012. **12**(1): p. 46.
30. Williams, A., Lloyd, A., Watson, L., and Rabe, K., *Cost of scheduled and unscheduled asthma management in seven European Union countries.* European Respiratory Review, 2006. **15**(98): p. 4-9.
31. Holtzer-Goor, K., et al., *Cost-effectiveness of including a nurse specialist in the treatment of urinary incontinence in primary care in the Netherlands.* PloS one, 2015. **10**(10): p. e0138225.
32. Lane, S., Molina, J., and Plusa, T., *An international observational prospective study to determine the cost of asthma exacerbations (COAX).* Respiratory medicine, 2006. **100**(3): p. 434-450.
33. Jaworski, R., Jankowska, E.A., Ponikowski, P., and Banasiak, W., *Costs of management of patients with coronary artery disease in Poland: the multicenter RECENT study.* Polskie Archiwum Medycyny Wewnetrznej, 2012. **122**(12): p. 599-607.
34. warszawaneurolog.pl. *Neurolog Warszawa*. 2017 [cited 2017 1 sep]; Available from: https://warszawaneurolog.pl/.

35. rehabilitacjanietubyc. *rehabilitacjanietubyc*. 2017 [cited 2017 1 sep]; Available from: http://rehabilitacjanietubyc.pl/en/home.
36. IMEDI. *IMEDI*. 2017 [cited 2017 1 sep]; Available from: http://imedi.pl/en/price-list/dietetics/.
37. tourmedica.co.uk. *tourmedica*. 2017 [cited 2017 1 sep]; Available from: http://www.tourmedica.co.uk/doctors/piotr-fryczkowski/#prices,retina-szpital-okulistyczny.
38. Ministério da Saúde. *Servico Nacional de Saude*. 2016 [cited 2017 1 Sep]; Available from: https://www.sns.gov.pt/.
39. Speets, A.M., Wolleswinkel, J.H., Forsgren, A., and Sobocki, P.A., *Use of medical resources and indirect costs of otitis media in Sweden.* Scandinavian journal of public health, 2011. **39**(2): p. 137-146.
40. Nordström, G., Danchenko, N., Despiegel, N., and Marteau, F., *Cost-effectiveness evaluation in Sweden of escitalopram compared with venlafaxine extended-release as first-line treatment in major depressive disorder.* Value in Health, 2012. **15**(2): p. 231-239.
41. Bachert, C., et al., *Cost-effectiveness of grass allergen tablet (GRAZAX®) for the prevention of seasonal grass pollen induced rhinoconjunctivitis–a Northern European perspective.* Clinical Experimental Allergy, 2007. **37**(5): p. 772-779.
42. Wellander, L., *Health and Economic Impact of Preventive Interventions for School Children Aimed to Improve Mental Health: Municipality perspective*, in *Department of Public Health and Caring Sciences*. 2014, Uppsala universitet.
43. Curtis, L.A. and Burns, A., *Unit costs of health and social care 2016*. 2016, University of Kent, Canterbury: Personal Social Services Research Unit.
44. Nasser, S., Vestenbaek, U., Beriot-Mathiot, A., and Poulsen, P., *Cost-effectiveness of specific immunotherapy with Grazax in allergic rhinitis co-existing with asthma.* Allergy, 2008. **63**(12): p. 1624-1629.
45. McCrone, P., et al., *Service use and costs for people with headache: a UK primary care study.* The journal of headache pain, 2011. **12**(6): p. 617.
46. Beecham, J., Chadwick, O., Fidan, D., and Bernard, S., *Children with severe learning disabilities: needs, services and costs.* Children society, 2002. **16**(3): p. 168-181.
47. NHS. *Reference costs*. 2017 [cited 2018 Dec 1]; Available from: https://data.gov.uk/dataset/489e83d2-7b05-41e1-b8f3-c06c1a8a9de4/health-trust-reference-costs-2016-17.
48. BMA. *Exploring the cost effectiveness of early intervention and prevention*. 2017 [cited 2017 1 Jan]; Available from: file:///C:/Users/u1373544/Downloads/Exploring-the-cost-of-early-intervention-ill-health-prevention.pdf.
